# Comprehensive fruit quality assessment and identification of aroma-active compounds in green pepper (*Capsicum annuum* L.)

**DOI:** 10.3389/fnut.2022.1027605

**Published:** 2023-01-10

**Authors:** Jing Zhang, Cheng Wang, Junwen Wang, Yan Yang, Kangning Han, Emily Patience Bakpa, Jing Li, Jian Lyu, Jihua Yu, Jianming Xie

**Affiliations:** ^1^College of Horticulture, Gansu Agricultural University, Lanzhou, China; ^2^State Key Laboratory of Aridland Corp Science, Gansu Agricultural University, Lanzhou, China

**Keywords:** pepper, texture, flavor, volatile compounds, odor activity value

## Abstract

The wrinkled pepper (*Capsicum annuum* L.) is a type of chili pepper domesticated in northwestern China, with a characteristic flavor. Fifteen wrinkled and four smooth-skinned pepper varieties were evaluated for morphology, texture, color, nutrients, capsaicinoids, and volatile compounds at the mature fruit stage. The sensory evaluation showed wrinkled pepper was superior to smooth pepper in texture, and it has a highly significant correlation (*p* < 0.01) with cuticle thickness, maximum penetrating force, lignin content, and moisture content. Citric acid was the major organic acid in peppers, accounting for 39.10–63.55% of the total organic acids, followed by quininic acid. The average oxalic acid content of smooth peppers was 26.19% higher than that of wrinkled peppers. The pungency of wrinkled pepper fruits ranged from 1748.9 to 25529.4 SHU, which can be considered slightly to very spicy, while the four smooth varieties ranged between 866.63 and 8533.70 SHU, at slightly to moderately spicy. A total of 199 volatile compounds were detected in the 19 pepper varieties. The average volatile content of wrinkled pepper was 39.79% higher than that of smooth pepper. Twenty-nine volatile compounds, including 14 aldehydes, four alcohols, three esters, three ketones, two furans, one pyrazine, one acid, and one phenol, contributed to the fragrance of peppers and could be regarded as aroma-active compounds, with 2-isobutyl-3-methoxypyrazine being the major contributor among the 19 pepper varieties. Wrinkled pepper can be confidently distinguished from smooth pepper and is of superior quality. The current findings outlined the major texture-related characteristics of pepper as well as the main aroma-active compounds, providing valuable information for pepper quality breeding and consumer guidelines.

## 1. Introduction

China’s agriculture has gradually entered a new era regarding quality standards. As the core of high-quality agricultural development, relevant characteristics extend beyond the content of nutrients beneficial to human health to also include intuitive sensory characteristics, such as flavor and texture (1). Since the green revolution, breeders have focused on improving plant resistance, yield, and firmness in variety selection, which are important for export and long-term storage (2). However, internal quality and texture have often been neglected, resulting in unsatisfactory product quality. With the development of the social economy and the improvement of living standards, the market has continuously raised quality requirements for horticultural products, and research has gradually shifted toward enhancing taste and functional nutrient content.

Capsicum (*Capsicum* spp.), which belongs to the Solanaceae family and genus Capsicum, originates from the tropical region of Latin America and is now cultivated worldwide (3). In 2019, the global area under pepper cultivation was 1.99 million hectares, of which China accounted for 40% of the total area and 50% of the total production (Food and Agriculture Organization of the United Nations (FAO) statistics; see URLs). China is now the world’s largest producer, consumer, and exporter of pepper. Hot pepper can be used as fresh food, a spice, or a colorant within the food industry, in addition to applications in medicine, the military, and other fields (4–6). The metabolic products of pepper fruit are considered an important source of compounds beneficial for human health (3, 7). A study previously characterized the phytochemical and nutrient composition of 147 dry pepper samples, revealing considerable variability between components and their levels among different Capsicum varieties (8). Pungency and aroma are important factors in the formation of pepper flavors. Capsaicinoids are only found in some species of the genus Capsicum, with their synthesis and biological regulation previously elucidated (9). These characteristics make pepper uniquely spicy and confer anticancer, analgesic, and weight-reducing properties (10, 11). However, quality parameters, such as aroma and texture, which receive great attention within the food industry, have not been subject to extensive research.

Odor, composed of various aromatic volatile metabolic components, is one of the most important factors affecting the quality of fruit and vegetable products, and is mainly determined by plant genetic factors (12). The cultivation of high-quality pepper varieties is inextricably linked to the collection of high-quality aroma germplasms, with basic research on the corresponding metabolites as well as the study of volatile organic compounds (VOC) composition being of great importance. However, owing to many pepper varieties and wide cultivation areas, there are considerable differences in the aroma types of peppers. Furthermore, aroma compounds are usually present at very low concentrations in foods and differ in their solubility, volatility, and stability at different temperatures and pH. Currently, over 300 VOCs have been identified in Capsicum fruits (13), and their combination within a given cultivar determines its unique flavor profile. In addition, the geographical origin, cultivar, and ripening processing also influence pepper aroma formation (14, 15). The characteristic volatile compounds in peppers remain unclear, despite the sophisticated analytical techniques currently available.

Northwest China has climatic conditions that are favorable for pepper cultivation. The pepper varieties produced there, such as “Longjiao” and “Sujiao,” have thin skin and thick flesh, and are spicy and slag-free. Because their fruit surfaces are wrinkled and have spiral lines, they are called “wrinkled” peppers. These are mainly grown in Gansu Province, being popular among the local people. However, there have been only a few studies on the physicochemical parameters of wrinkled peppers, with research on flavor formation still very limited. Therefore, the basis for high-quality horticultural production is to explore the mechanisms that determine the excellent taste quality of pepper varieties. Considering the above information, the main objective of this study was to evaluate the texture and flavor characteristics of wrinkled peppers by comparing the chemical constituents of 19 pepper varieties, which would provide a basis for further research on the quality formation mechanisms in peppers.

## 2. Materials and methods

### 2.1. Plant materials

**FIGURE 1:**
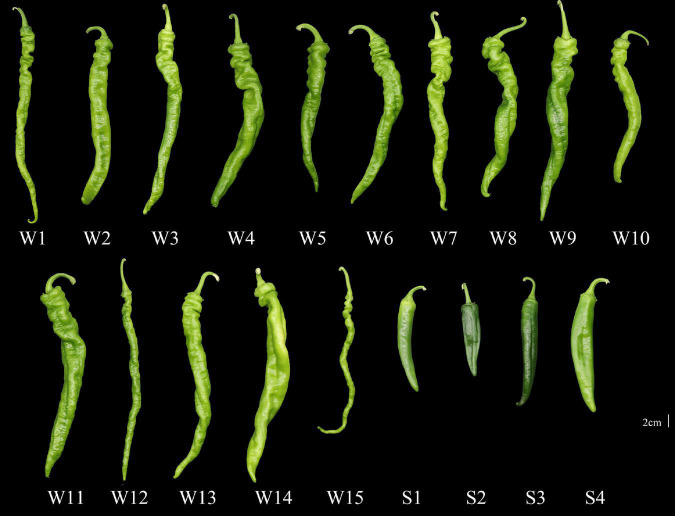
Pepper varieties used in the present study. W1, Fengshouxianjiao; W2, Sujiao 8; W3, Qingan 7; W4, Sujiao 18; W5, Longjiao 2; W6, Longjiao 10; W7, Longjiao 11; W8, Huamei 105; W9, 37–124; W10, 3F*106; W11, Tianjiao 20; W12, Tianjiao 23; W13, Hangjiao 2; W14, Hangjiao 8; W15, NO.171; S1, NO.212; S2, NO.221; S3, Sujiao 9; S4, HJF42.

On 8 April 2021, 19 varieties of pepper seedlings (Fengshouxianjiao, Sujiao 8, Qingan 7, Sujiao 18, Longjiao 2, Longjiao 10, Longjiao 11, Huamei 105, 37–124, 3F*106, Tianjiao 20, Tianjiao 23, Hangjiao 2, Hangjiao 8, NO.171, NO.212, NO.221, Sujiao 9, and HJF42) grown on plastic trays were transplanted to the greenhouse in Hekou Town, Xigu District, Lanzhou city, Gansu, China (35°85′N, 104°12′E). The varieties NO.212, NO.221, Sujiao 9, and HJF42 were characterized by a smooth pericarp, whereas the other pepper varieties were characterized by different degrees of wrinkles in the pericarp. Pepper plants were grown under the same conditions and daily care procedures. For harvesting, mature green (commodity maturity stage) pepper fruits of the same size without visible defects or diseases were randomly selected from the plastic greenhouse and immediately transported to the laboratory. The 19 varieties used in this study are shown in Figure 1. The average weight of the fruits at the commodity maturity stage are listed in Supplementary Table 1.

### 2.2. Analysis of pepper fruit physical parameters

Six fruits of each variety were randomly selected to measure fruit length and width using a tape measure and a vernier caliper, respectively. The fruit shape index was calculated from the ratio of fruit length to width. We used a TMS-Pro texture analyzer testing frame (TMS-PRO, FTC, USA) to measure the fruit’s maximum penetrating force and shearing force, with three measurement positions for each fruit. The fresh weight was determined, and peppers were then dried in an oven at 80°C to a constant weight, whereafter dry weight was determined and the moisture content calculated. The fruit-specific gravity was obtained by calculating the ratio of fresh weight to the volume of the pepper. Pericarp thickness was measured using a Vernier caliper, and three fruit positions were selected. To measure cuticle thickness, we cut pericarp sections from the fruit and immediately fixed the tissue in a formalin-acetic acid alcohol buffer. Subsequently, the samples were dehydrated with a gradient ethanol series, immersed in paraffin, embedded, sectioned, and stained. Sections were observed under a microscope, and the thickness was analyzed with Image J software (16).

### 2.3. Analysis of cellulose, lignin, and protopectin content

To determine cellulose content, 0.1 g dried pepper powder was homogenized in 5 mL nitric and acetic acids. After boiling for 25 min, samples were centrifuged. The clean pellet was mixed with 10 mL of 0.5 N potassium dichromate and 8 mL of concentrated sulfuric acid. The solution was boiled at 100°C for 10 min. While the solution was cooling, two to three drops of the test ferrous indicator were added and titrated with 0.1 N ferrous ammonium sulfate (17).

To determine lignin content, the 0.1 g dried pepper powder was homogenized in 10 mL of 1% acetic acid, vibrated for 5 min, and then centrifuged. Acetic acid (1%) and acetone were used to wash pellets. The clean pellet was mixed with 3 mL sulfuric acid for 16 h. The volume was increased to 10 mL with pure water. The pellet was obtained by adding 0.5 mL of barium chloride (10%) and then mixing with sulfuric acid-potassium dichromate solution. As the solution cooled, two-three drops of test ferrous indicator were added and then titrated with 0.1 N ferrous ammonium sulfate (17).

Fresh pepper (0.2 g) was extracted with 0.01% PBS buffer, and protopectin content was determined based on the OD at 450 nm, using an enzyme-linked immunosorbent assay (ELISA) kit (Guduo Institute of Biological Technology, Shanghai, China) as per the manufacturer’s instructions.

### 2.4. Sensory assessment of pepper texture

An affective test was conducted to assess the texture preferences and acceptability of 19 types of peppers, using a slightly modified procedure (18). Panelists aged 21 to 52 years were invited from the College of Horticulture, Gansu Agricultural University. Fresh peppers were cut open and randomly presented to the panelists. They were asked to pay attention to how difficult it was to bite off the skin of the peppers with their front teeth and remove the residue with their molars after they had fully chewed. Testing was conducted in a clean, quiet, odor-free room, and filtered water was provided between samples to cleanse the palate. Based on a well-matched 9-point hedonic scale, ratings were presented as follows: 9 = exceptionally like, 8 = very like, 7 = moderately like, 6 = like, 5 = neutral, 4 = moderately dislike, 3 = dislike, 2 = very dislike, and 1 = exceptionally dislike. The sensory assessment was conducted three times, with 92 trained panelists participating.

### 2.5. Analysis of color difference

Color difference analysis of the peppers was performed using a portable chromatic aberration meter (CR-10 Plus, Konica Minolta, Inc., Tokyo, Japan). Color difference indicators L*, a*, and b* were calculated, representing lightness (L*), red/green (a*), and yellow/blue (b*), respectively (19). Measurements were performed on the central part of each fruit.

### 2.6. Analysis of vitamin C and soluble sugar

Six fresh pepper fruits were washed with distilled water and rapidly homogenized using a homogenizer. The vitamin C content was measured using the 2,6-dichloroindophenol staining method in conjunction with a UV-1780 spectrophotometer (Shimadzu Instruments Co., Ltd., Suzhou, China) at 500 nm (20). Total soluble sugar content was measured using the anthrone method at 620 nm (21). All analyses were performed in triplicate.

### 2.7. Analysis of organic acids

For organic acids analysis, 0.5 g of homogenized pepper was transferred to a 25 mL volumetric flask containing ultrapure water to keep the volume constant, shaken well, and the mixture was then centrifuged at 10,000 r min^–1^ for 10 min at 4°C. 2 mL of supernatant was extracted and filtered through a 0.22 μm water system, whereafter the filtrate was used for liquid chromatography. The measuring instrument was a Waters Acquity Arc UHPLC system (Waters, MA, USA), and the chromatographic column was an Atlantis T3 column (150 × 4.6 mm, 3 μm). The following settings were applied: detection wavelength: 210 nm, mobile phase: 0.2 mmol L^–1^ dihydrogen phosphate sodium, isocratic elution, flow rate 1.2 mL min^–1^, column temperature 30°C, injection volume was 5 μL. All assays were performed in triplicate.

### 2.8. Analysis of pungency

After the pedicles were removed, the 60°C-dried peppers of each variety were crushed into powder and thoroughly mixed to determine capsaicin and dihydrocapsaicin contents. Methanol-tetrahydrofuran (1:1) was added to 1 g of the powder sample for extraction. The mixture was shaken for 50 min at 60°C and then concentrated to 10–20 mL using a rotary evaporator. When the volume was fixed at 50 mL with methanol-tetrahydrofuran (1:1), the mixture was filtered through a Millipore membrane with a pore size of 0.22 μm. The chromatographic system consisted of a Waters 2,998 (America) with a ZORBAX Eclipse Plus C_18_ column (250 × 4.6 mm, 5.0 μm). The absorbance at 280 nm was measured using a Shimadzu UV-VIS 1700 spectrophotometer. Methanol-H_2_O (65:35) was used as the mobile phase at a flow rate of 1.0 mL min^–1^. The injection volume for each sample was 5 μL. Methanol and tetrahydrofuran were of HPLC grade, and ultrapure water was used for HPLC analysis (22). A series of mixed standard solutions were prepared with capsaicin (Sigma, USA) and dihydrocapsaicin (Sigma, USA). After determining the contents of capsaicin and dihydrocapsaicin, the pungency was measured in Scoville heat units (SHU), calculated as per the following equation (4):


(1) $$\text{SHU}=\left(X_{1}+X_{2}\right)\times \left(16.1\times 10^{3}\right)+\left(X_{1}+X_{2}\right)/90\%$$

$$\;\times 10\%\times \left(9.3\times 10^{3}\right)$$

where *X*_1_ and *X*_2_ represent capsaicin and dihydrocapsaicin content, respectively (mg g^–1^), 90% is the ratio of capsaicin and dihydrocapsaicin in capsaicinoids, 16.1 × 10^3^ for capsaicin and dihydrocapsaicin in SHU, 9.3 × 10^3^ for the others capsaicinoids in SHU.

### 2.9. Evaluation of volatile compounds

Gas chromatography-mass spectrometry (GC-MS), in combination with aroma thresholds, was used to comprehensively study the aroma compounds in different cultivated pepper varieties. A homogenized fresh pepper sample (8 g) was weighed and placed in a headspace vial (25 mL). 10 μL of 2-octanol (8.82 mg L^–1^) was added as the internal standard. Sodium chloride (1.5 g) and a magnetic stirring rotor were added, whereafter, the plastic cap was quickly sealed with polytetrafluoroethylene silicone septa. Samples were incubated for 10 min at 50°C in a heating bath. The volatile compounds were extracted using an SPME fiber inserted into a vial for 20 min. The fiber needle was immediately inserted into the injection port for 3 min for desorption (23, 24).

The volatile compounds were isolated and identified using a gas chromatographer (Agilent model 7890 B) equipped with a mass spectrometer detector (Agilent 7000C) and a DB-WAX elastic quartz capillary column (20 m × 0.18 mm, 0.18 μm) with helium (≥ 99.999% purity) at a flow rate of 1.0 mL/min as the carrier gas. Spitless injection mode was applied when the volatiles were introduced at 230°C. The slightly modified temperature program was first held at 40°C for 5 min, then increased to 100°C at a rate of 5°C/min, then increased to 150°C at 8°C/min, and finally increased to 210°C at 10°C/min for 5 min (25). The mass spectrometer was operated per the electron impact method with an ionization energy of 70 eV and a source temperature of 230°C. The full scan mode was used for mass spectrometry with a mass range of 33–500 m/z. The filament current and quadrupole temperature were 150 μA and 250°C, respectively.

### 2.10. Qualitative and quantitative analysis of volatile compounds

After GC-MS analysis, each volatile compound was analyzed using the automatic deconvolution system of the computer workstation and the mass spectrometry library (NIST 2014) based on its mass fragmentation pattern from the spectral database. Only compounds with an MS match greater than 70% were considered. The concentration of each compound in pepper was calculated using the internal standard method and the following formula (26):


(2) C⁢(μ⁢g/kg)=(A1/A2)×(M1/M2)×1000

A1 and A2 are the component areas of the detected compositions and internal standard, respectively; M1 and M2 are the internal standard and sample amounts, respectively. Average values were calculated from three replicates.

Using the aroma threshold values for volatile compounds reported in the literature, the aroma activity values (OAV) were calculated, and compounds with OAV > 1 were considered as the aroma-active compounds of pepper. When the OAV value was greater than 1, the volatiles were considered aroma-active compounds. As shown in the formula, OT is the aroma threshold value for volatile compounds, and C is the concentration of volatile compounds in pepper fruit (μg kg^–1^) (27).


(3) OAV=C/OT

### 2.11. Calculation of the comprehensive membership function

The positive and negative index membership function values were calculated using Equations (4) and (5). where Xij(u) is the membership function value of the jth index of the ith breed, Xij is the measured value of the jth index of the ith breed, Xjmin is the minimum value of the jth index in the tested variety, and Xjmin is the maximum value of the jth index among the tested varieties (28).


(4) Xij⁢(u)=(Xij-Xjmin)/(Xjmax-Xjmin)

(5) Xij⁢(u)=1-(Xij-Xjmin)/(Xjmax-Xjmin)

The value of the comprehensive membership function was calculated as follows:


(6) $$\text{Xi}\,\left(\text{u}\right)\,\left[\sum_{i=1}^{\text{n}}\text{Xij}\,\left(\text{u}\right)\right]/\text{n}$$

### 2.12. Statistical analysis

Analysis of variance (ANOVA) was performed using SPSS version 19.0 (IBM, Chicago, USA). Data are expressed as mean ± standard error of replicates for each treatment. Statistical differences between treatments were compared using Duncan’s multiple range test, and differences were considered statistically significant at *p* < 0.05. Origin 2021 software was used to generate figures and carry out principal component analysis (PCA).

## 3. Results

### 3.1. The physical indicators of pepper fruit

The fruit moisture content, shape index, maximum penetrating and shearing forces, pericarp thickness, and cuticle thickness of peppers are presented in Table 1. The fruit shape index of Tianjiao 23, Fengshouxianjiao, and NO.171 were significantly higher than those of the other varieties, exhibiting a thin and long appearance. The specific gravity of Fengshouxianjiao was the highest (0.77). In addition, there were no significant differences among the other 19 varieties, although the fruit types differed. The maximum penetrating force of smooth pepper was higher than that of the wrinkled pepper, except for Qingan 7. The NO.212 variety had the highest mean maximum penetrating force (11.77 N), followed by HJF42 and Sujiao 9. For shearing force, the Huamei 105, Hangjiao 8, NO.212, and 37–124 varieties tended to have higher values than the other varieties. The pericarp thickness of Sujiao 8 was significantly higher than for other varieties, while NO.171 and Tianjiao 23 had the lowest thicknesses and were significantly thinner compared to the other varieties. The values for the thickness of the wrinkled pepper cuticle were 8.25–15.60 μm and thus had a significantly lower thickness than smooth varieties. The moisture content of fresh peppers ranged from 88.72 to 93.46%, with NO.221 having the lowest value, followed by Sujiao 9, while 3F × 106 had the highest moisture content.

**TABLE 1:** Fruit traits of the evaluated 19 pepper fruit varieties.

Varieties	Texture score	Fruit shape index	Specific gravity	Maximum penetrating force (*N*)	Maximum shearing force (*N*)	Pericarp thickness (mm)	Cuticle thickness (um)	Moisture content (%)
W1	6.05 ± 0.17def	18.01 ± 0.76b	0.77 ± 0.04a	5.37 ± 0.26e	46.89 ± 3.46def	1.96 ± 0.1f	11.55 ± 1.01fgh	91.75 ± 0.35bcde
W2	6.80 ± 0.31bcd	9.56 ± 0.37defg	0.60 ± 0.03bcdefg	5.24 ± 0.3ef	69.68 ± 3.16abc	3.51 ± 0.24a	8.25 ± 0.19h	92.77 ± 0.39abc
W3	7.11 ± 0.43abcd	10.23 ± 0.36def	0.59 ± 0.02cdefg	7.42 ± 0.38cd	56.65 ± 4.59bcd	2.18 ± 0.11def	9.78 ± 0.15gh	92.06 ± 0.2abcd
W4	6.97 ± 0.22bcd	9.62 ± 0.54defg	0.54 ± 0.01efg	4.45 ± 0.26ef	71.81 ± 7.96abc	2.14 ± 0.15def	9.95 ± 0.12gh	93.02 ± 0.26ab
W5	6.62 ± 0.13cde	10.14 ± 0.36def	0.68 ± 0.06bc	5.37 ± 0.27e	63.81 ± 2.54abcd	1.99 ± 0.05ef	16.96 ± 2.39c	91.65 ± 0.7bcde
W6	7.12 ± 0.24abcd	7.22 ± 0.31ijk	0.63 ± 0.01bcdef	4.64 ± 0.28ef	61.09 ± 1.95abcd	2.26 ± 0.09cdef	12.68 ± 1.08defg	92.89 ± 0.16ab
W7	7.54 ± 0.28abc	10.66 ± 0.47de	0.63 ± 0.05bcde	5.03 ± 0.24ef	65.14 ± 4.42abcd	2.09 ± 0.07def	11.81 ± 0.72efgh	92.70 ± 0.14abc
W8	7.10 ± 0.23abcd	9.23 ± 0.61efgh	0.55 ± 0.01efg	5.44 ± 0.29e	81.03 ± 6.9a	2.43 ± 0.08bcd	9.40 ± 0.26gh	92.96 ± 0.23ab
W9	6.95 ± 0.4bcd	8.07 ± 0.62ghi	0.58 ± 0.01defg	4.95 ± 0.27ef	77.15 ± 2.32ab	2.29 ± 0.09cdef	15.60 ± 1.46cde	90.55 ± 1.05def
W10	6.71 ± 0.23bcde	11.28 ± 0.5d	0.70 ± 0.06ab	5.11 ± 0.24ef	69.88 ± 4.38abc	2.21 ± 0.17def	15.93 ± 0.66cd	93.46 ± 0.8a
W11	8.37 ± 0.28a	8.60 ± 0.4fghi	0.55 ± 0.01efg	4.55 ± 0.25ef	48.45 ± 4.06def	2.69 ± 0.16b	11.03 ± 0.44gh	93.14 ± 0.16ab
W12	6.81 ± 0.46bcd	20.36 ± 0.93a	0.65 ± 0.02bcd	4.09 ± 0.21ef	34.12 ± 3.84ef	1.48 ± 0.08g	8.49 ± 0.18gh	91.00 ± 0.15def
W13	6.78 ± 0.13bcd	10.65 ± 0.57de	0.60 ± 0.01bcdefg	5.37 ± 0.55e	63.24 ± 3abcd	2.17 ± 0.09def	9.28 ± 0.59gh	91.81 ± 0.41bcd
W14	8.07 ± 0.18ab	7.71 ± 0.36hij	0.53 ± 0.01fg	6.83 ± 0.62d	80.50 ± 8.43a	2.60 ± 0.1bc	15.28 ± 0.32cdef	93.43 ± 0.12a
W15	6.49 ± 0.08cde	13.76 ± 1.48c	0.65 ± 0.02bcd	4.76 ± 0.53ef	29.66 ± 2.45f	1.55 ± 0.14g	10.39 ± 0.22gh	91.33 ± 0.36cdef
S1	4.86 ± 0.62fg	7.58 ± 0.2hij	0.66 ± 0.02bcd	11.77 ± 0.45a	73.03 ± 6.85abc	2.47 ± 0.08bcd	31.52 ± 0.59b	90.27 ± 0.75ef
S2	4.29 ± 0.58g	3.68 ± 0.16l	0.52 ± 0.02g	7.06 ± 0.32d	52.57 ± 1.93cde	2.20 ± 0.1def	38.76 ± 0.8a	88.72 ± 0.54g
S3	4.55 ± 0.44g	6.16 ± 0.24jk	0.67 ± 0.04bcd	8.31 ± 0.46c	73.74 ± 9.01abc	2.37 ± 0.08bcde	30.38 ± 0.64b	89.85 ± 0.42fg
S4	5.43 ± 1.11efg	5.52 ± 0.18k	0.65 ± 0.02bcd	9.64 ± 0.6b	80.74 ± 17.68a	2.48 ± 0.06bcd	31.13 ± 4.19b	90.84 ± 0.27def

Data represent the mean ± SE. Different lowercase letters indicate statistical significance by Duncan’s multiple range Test (p < 0.05). W1, Fengshouxianjiao; W2, Sujiao 8; W3, Qingan 7; W4, Sujiao 18; W5, Longjiao 2; W6, Longjiao 10; W7, Longjiao 11; W8, Huamei 105; W9, 37–124; W10, 3F*106; W11, Tianjiao 20; W12, Tianjiao 23; W13, Hangjiao 2; W14, Hangjiao 8; W15, NO.171; S1, NO.212; S2, NO.221; S3, Sujiao 9; S4, HJF42.

### 3.2. Cellulose, lignin, and protopectin content in pepper fruit

**FIGURE 2:**
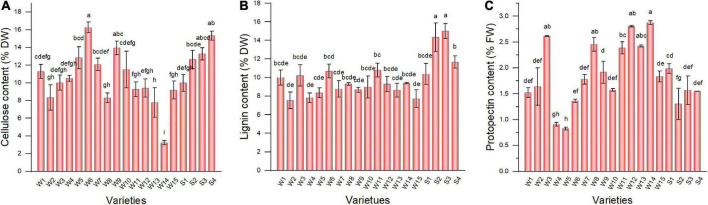
The cellulose **(A)**, lignin **(B)**, and protopectin **(C)** content of pepper varieties. Data are presented as mean ± SE. Different lowercase letters indicate statistical significance according to Duncan’s multiple range test (*p* < 0.05). W1, Fengshouxianjiao; W2, Sujiao 8; W3, Qingan 7; W4, Sujiao 18; W5, Longjiao 2; W6, Longjiao 10; W7, Longjiao 11; W8, Huamei 105; W9, 37–124; W10, 3F*106; W11, Tianjiao 20; W12, Tianjiao 23; W13, Hangjiao 2; W14, Hangjiao 8; W15, NO.171; S1, NO.212; S2, NO.221; S3, Sujiao 9; S4, HJF42.

The pepper varieties exhibited significant differences in cellulose (Figure 2A), lignin (Figure 2B), and protopectin (Figure 2C) contents in the fruit. Except for Hangjiao 8, the cellulose content of all tested pepper varieties was >5%. Longjiao 10, HJF42, and 37–124 had higher cellulose content (16.27, 15.33, and 13.95%, respectively). The lignin content of tested varieties ranged from 7.55 to 15.00%. S3 had the highest lignin content, followed by NO.221 (14.35%). Sujiao 8 had the lowest lignin content. The protopectin content of the 19 varieties ranged from 0.83 to 2.87%, which was the highest in Hangjiao 8, which was contrary to the cellulose results.

### 3.3. Sensory assessment pepper texture

The mean sensory assessment scores of pepper texture are shown in Table 1. Scores given by panelists presented a clear difference between wrinkled and smooth pepper in texture, with an average score between 6.05 and 8.37 for the wrinkled pepper varieties and 4.29 to 5.43 for smooth pepper varieties. Tianjiao 20 had the highest sensory score, without significant differences, compared to Qingan 7, Longjiao 10, Longjiao 11, Huamei 105, and Hangjiao 8.

### 3.4. Color difference of pepper fruit

**FIGURE 3:**
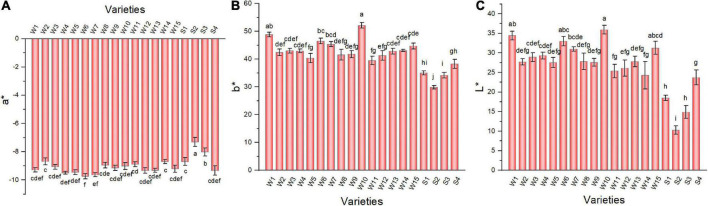
The a* **(A)**, b* **(B)**, and L* values **(C)** of pepper varieties. Data are presented as mean ± SE. Different lowercase letters indicate statistical significance according to Duncan’s multiple range test (*p* < 0.05). W1, Fengshouxianjiao; W2, Sujiao 8; W3, Qingan 7; W4, Sujiao 18; W5, Longjiao 2; W6, Longjiao 10; W7, Longjiao 11; W8, Huamei 105; W9, 37–124; W10, 3F*106; W11, Tianjiao 20; W12, Tianjiao 23; W13, Hangjiao 2; W14, Hangjiao 8; W15, NO.171; S1, NO.212; S2, NO.221; S3, Sujiao 9; S4, HJF42.

Color difference indicators also differed among the pepper varieties (Figure 3). The a* values of the 19 pepper varieties were negative (negative values represent green color), with W6 having the lowest value and showing a greener appearance. The b* value (positive values indicated yellow) of W10 was the highest; thus, it was more yellow than other varieties. The L* values of three smooth peppers, including NO.212, NO.221, and Sujiao 9 varieties were significantly lower than that of the other varieties.

### 3.5. Vitamin C and soluble sugar in pepper fruit

**FIGURE 4:**
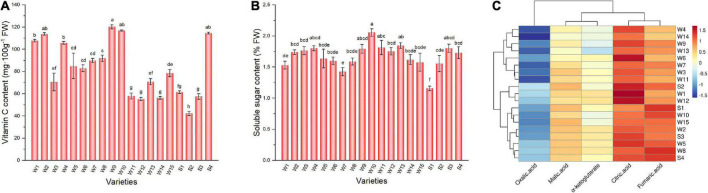
Vitamin C **(A)**, soluble sugar **(B)**, and organic acid composition **(C)** in peppers. Data in Panels **(A,B)** are presented as the mean ± SE. Different lowercase letters indicate statistical significance by Duncan’s multiple range test (*p* < 0.05). The colored areas in panel **(C)** correspond to the content of acids from low (blue) to high (red), the data has been log_2_-transformed and standardized. Euclidean distance and average linkage were used to construct the clustering of acids and varieties. W1, Fengshouxianjiao; W2, Sujiao 8; W3, Qingan 7; W4, Sujiao 18; W5, Longjiao 2; W6, Longjiao 10; W7, Longjiao 11; W8, Huamei 105; W9, 37–124; W10, 3F*106; W11, Tianjiao 20; W12, Tianjiao 23; W13, Hangjiao 2; W14, Hangjiao 8; W15, NO.171; S1, NO.212; S2, NO.221; S3, Sujiao 9; S4, HJF42.

Green peppers are generally rich in vitamin C. The vitamin C content varied from 42.13 to 120.24 mg 100 g^–1^ among varieties (Figure 4A). Varieties Sujiao 8, 37–124, 3F × 106, and HJF42 contained more than 110 mg 100 g^–1^ vitamin C, and there was no significant difference among these varieties, while the vitamin C content of variety NO.221 was the lowest among all varieties studied. There were differences in soluble sugar content among varieties (Figure 4B). More than 2% soluble sugar content was observed in 3F × 106 (2.06%) pepper fruits, whereas variety NO.212 (1.16%) had the lowest soluble sugar content.

### 3.6. Organic acid composition and content of pepper fruit

Organic acids are essential components of pepper, contributing to nutritional value and taste (29). The total organic acid content determined herein ranged from 5.83 to 11.32 mg/g FW, with Fengshouxianjiao having the highest level and Hangjiao 8 having the lowest level of total organic acids (Figure 4C). Citric acid is the main organic acid in these samples, contributing 24.56–49.53% of total organic acid content and ranging from 1.82 mg/g to 5.60 mg g^–1^ FW. Fumaric acid was the second most abundant organic acid in the 19 varieties of pepper, amounting to 18.84–33.78% of the total organic acid content and ranging from 1.48 to 3.71 mg⋅g^–1^ FW. Malic acid was the third most abundant organic acid, accounting for 12.71–21.61% of the total organic acids. Its content ranged from 0.99 to 1.88 mg g^–1^ FW. The α-ketoglutarate detected in pepper fruit accounted for only 10.44–16.80% of the total organic acid content, ranging from 0.70 to 1.43 mg g^–1^ FW. Oxalic acid was detected at less than 8%, with contents below 0.7 mg g^–1^ FW in tested peppers.

### 3.7. Capsaicinoid content and pungency of pepper fruit

**FIGURE 5:**
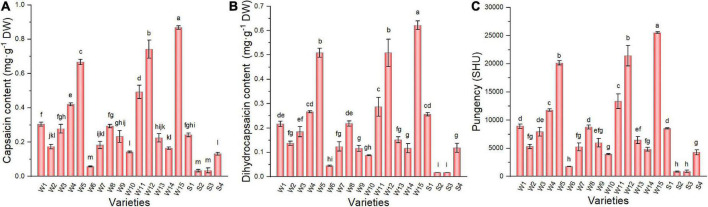
The capsaicin content **(A)**, dihydrocapsaicin content **(B)**, and pungency **(C)** of pepper fruits. Data are presented as mean ± SE. Different lowercase letters indicate statistical significance according to Duncan’s multiple range test (*p* < 0.05). W1, Fengshouxianjiao; W2, Sujiao 8; W3, Qingan 7; W4, Sujiao 18; W5, Longjiao 2; W6, Longjiao 10; W7, Longjiao 11; W8, Huamei 105; W9, 37–124; W10, 3F*106; W11, Tianjiao 20; W12, Tianjiao 23; W13, Hangjiao 2; W14, Hangjiao 8; W15, NO.171; S1, NO.212; S2, NO.221; S3, Sujiao 9; S4, HJF42.

The results for capsaicin (Figure 5A) and dihydrocapsaicin (Figure 5B) in the pepper varieties studied were considerably different. Capsaicin content was higher, and the trend among varieties was consistent with the results for dihydrocapsaicin. The NO.171 variety had the highest content of capsaicin (0.87 g kg^–1^) and dihydrocapsaicin (0.62 g kg^–1^), which were 25.62 and 37.24 times greater than that of the S2 variety, respectively. The Scoville organic test, invented by Scoville in 1912, is a subjective measure of chili peppers hotness. Depending on the capsaicin and dihydrocapsaicin content, pungency (Figure 5C) varied over a wide range among the pepper varieties (25525–866.63 SHU). The two highest pungency values were determined for wrinkled varieties (NO.171 and Tianjiao 23), while the two lowest values were determined for smooth varieties (NO.221 and Sujiao 9).

### 3.8. Volatile compounds in pepper fruit

**FIGURE 6:**
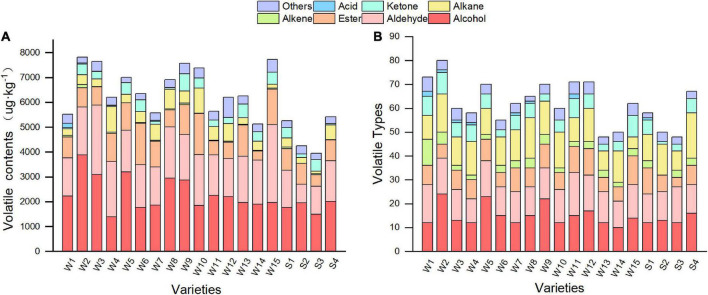
Content **(A)** and type **(B)** of volatile compounds in pepper. W1, Fengshouxianjiao; W2, Sujiao 8; W3, Qingan 7; W4, Sujiao 18; W5, Longjiao 2; W6, Longjiao 10; W7, Longjiao 11; W8, Huamei 105; W9, 37–124; W10, 3F*106; W11, Tianjiao 20; W12, Tianjiao 23; W13, Hangjiao 2; W14, Hangjiao 8; W15, NO.171; S1, NO.212; S2, NO.221; S3, Sujiao 9; S4, HJF42.

In this study, 199 different volatile compounds were detected in the 19 varieties of pepper at the green maturity stage using the headspace solid-phase microextraction (HS-SPME)/GC-MS method. These included 55 alcohols, 30 aldehydes, 22 esters, 16 ketones, 36 alkenes, 18 alkanes, 6 acids, and 16 other volatile compounds, such as furan and aromatic hydrocarbons. The type and number of volatile compounds varied among different pepper varieties. The greatest number of volatile compounds were detected in Sujiao 8, with 79, while the other varieties generally contained between 47 and 71 volatile compounds (Figure 6A). Alcohols, alkenes, and aldehydes are the main compound classes in pepper fruits. To evaluate the differences in volatile content within the airspace of peppers, the relative content of volatiles was estimated by adding a measured amount of an internal standard to the peppers, and the identified compounds were quantified, as shown in Supplementary Table 1. The total volatile compound content was lowest in variety Sujiao 9 (3953.77 μg⋅kg^–1^), whereas variety Sujiao 8 had the highest volatile compound content (7824.05 μg⋅kg^–1^) (Figure 6B). The average volatile compound content of smooth pepper was 4727.19 μg⋅kg^–1^, whereas that of wrinkled pepper was 6608.14 μg⋅kg^–1^. The relative content of alcohols and aldehydes accounted for 22.55–49.75% and 17.38–40.48% of the total volatile compound content, respectively, and were higher than other compound classes.

### 3.9. Analysis of aroma-active compounds in pepper

Table 2 shows the VOC names, odor descriptors, perception threshold, and OAV for the 19 pepper varieties. The OAVs were determined based on reference aroma threshold values (26, 30, 31). There were 29 volatiles with OAVs greater than 1 that played a role in pepper odor formation. Among these, 2-methoxy-3-(2-methylpropyl)-pyrazine had the highest OAV (81713.36-164461.40) and contributed greatly to the green pepper fragrance of all varieties. Otherwise, hexanal, (E)-2-hexenal, methyl salicylate, nonanal, (E)-3-hexen-1-ol, and (E, E)-2,4-decadienal, with an OAV greater than 1, were present in all samples and are indispensable to pepper fragrance. While decanal was not detected in Sujiao 18, it has a high OAV value (168.02–522.35) among the other 18 varieties, contributing a strong citrus, sweet, and waxy aroma. The odor of 2-ethyl-furan was defined as beany, ethereal cocoa, and bready, contributing to aroma formation in the 18 pepper varieties (all but Tianjiao 20), with an OAV that ranged from 3.34 to 30.17. β-Ionone has a floral and woody aroma. It was not detected in NO.221 and Sujiao 9, but had high OAV values of between 361.22 and 5010.53 in the other 17 varieties. Linalool imparts a floral, woody, and citrus-like aroma to pepper. Except for in NO.212 (OAV = 0.54) and Sujiao 9 (OAV = 0.81), the OAV of linalool ranged from 1.22 to 9.97 among other pepper varieties. Ethyl salicylate is described as having a sweet, floral, and spicy flavor. It was detected in all varieties, but had an OAV value greater than 1 only in NO.171, which was the most pungent variety. The OAVs of five compounds, including 2-hexenal, undecanal, 2,3-octanedione, pentanoic acid, and 2-methoxy-phenol, were greater than 1 in only one variety.

**TABLE 2:** Odor activity values of 29 volatile compounds in 19 pepper varieties.

Compounds name	Odor description	Odor threshold	Odor activity values (OAVs)																		
		**μg kg^–1^**	**W1**	**W2**	**W3**	**W4**	**W5**	**W6**	**W7**	**W8**	**W9**	**W10**	**W11**	**W12**	**W13**	**W14**	**W15**	**S1**	**S2**	**S3**	**S4**
1-octen-3-ol	Mushroom, earthy, chicken	1	2.05	2.89	7.17	-	8.16	-	-	-	1.08	8.50	0.00	-	-	-	8.21	-	-	5.21	-
1-Hexanol	Fruity, sweet, green	5.7	24.68	41.35	85.85	-	75.02	83.21	-	81.18	54.72	23.67	42.84	16.37	53.87	48.43	-	42.09	-	-	46.33
Linalool	Citrus, floral woody	6	1.60	4.05	2.83	1.71	5.11	6.41	3.89	2.98	6.11	6.62	3.87	2.46	3.95	3.95	9.97	0.54	1.22	0.81	2.36
(E)-3-hexen-1-ol	Green, floral, oily	70	5.57	5.40	2.86	5.92	7.03	5.96	6.08	9.42	5.17	3.92	9.99	8.97	5.92	10.53	5.20	4.66	4.96	7.55	5.42
(E,E)-2,4-Non-adienal	Fatty, melon, chicken	0.09	-	466.95	84.47	-	-	-	-	-	-	-	-	-	-	-	-	-	-	-	-
Hexanal	Fresh green fruity	4.5	67.35	258.65	430.65	309.95	203.35	213.56	203.93	304.22	289.68	298.96	254.71	223.56	213.92	201.81	481.99	137.23	103.74	56.31	142.73
*Trans*-2-hexenal	Green banana	17	27.03	28.95	36.43	45.66	27.23	35.12	22.80	27.38	19.69	33.25	14.75	17.08	38.91	41.14	41.55	39.15	9.59	36.89	50.56
β-cyclocitral	Herbal sweet, fruity	5	1.65	1.10	0.94	0.48	0.56	-	0.71	0.73	0.80	0.60	0.43	0.26	0.67	0.71	1.10	0.61	0.40	0.33	0.34
(E)-2-Octenal	Fresh, fatty, green	3	17.38	11.41	19.43	-	27.05	-	14.38	-	20.27	-	4.93	2.92	18.82	10.02	-	10.29	5.42	10.35	9.49
3-methyl-Butanal	Chocolate, Peach, fatty	0.25	-	-	-	-	-	-	-	-	-	-	-	-	-	-	7.77	-	6.05	-	-
(E)-2-Non-enal	Fatty, green, cucumber	0.4	171.35	-	-	-	130.17	-	234.29	223.13	-	-	4.66	-	37.58	-	-	-	-	5.32	-
2-Hexenal	Sweet, fruity, green	17	18.41	-	-	-	-	-	-	-	-	-	0.60	-	-	-	-	-	-	-	-
Heptanal	Fresh, green, herbal	2.9	4.39	3.41	3.35	1.86	3.95	5.29	0.49	4.08	3.21	5.01	0.85	0.25	4.17	0.07	4.92	0.77	2.13	0.70	0.90
Decanal	Sweet, waxy, citrus	0.1	283.15	522.35	321.63	-	245.87	228.32	233.30	274.31	406.96	263.08	296.76	428.28	372.68	316.50	455.30	456.12	215.62	168.02	247.87
Undecanal	Floral, citrus	5	1.10	0.77	0.55	-	-	0.13	-	-	-	-	-	-	-	-	-	-	-	0.51	-
Benzeneacetaldehyde	Floral green sweet	4	3.03	1.99	-	0.77	-	-	-	-	-	0.79	1.67	1.07	-	-	4.32	0.95	2.18	1.63	0.77
Non-anal	Rose, orange, fatty	1	30.84	37.81	22.04	10.25	21.82	30.74	14.46	18.05	30.37	19.20	17.57	44.45	30.84	27.77	27.63	17.77	15.52	14.87	27.69
(E,E)-2,4-decadienal	Oily, cucumber, citrus	0.07	196.89	187.84	1185.41	344.67	246.00	319.69	475.42	253.76	551.29	246.29	364.86	282.29	589.02	445.78	435.95	344.11	117.17	106.43	97.65
Methyl salicylate	Wintergreen mint	40	1.92	2.93	2.21	2.74	2.98	1.65	2.60	2.52	2.47	3.17	1.07	1.06	1.55	2.44	5.01	3.91	4.18	2.22	1.90
Ethyl salicylate	Sweet, floral, spicy	84	0.19	0.33	0.26	0.32	0.32	0.33	0.45	0.40	0.33	0.44	0.24	0.20	0.35	0.34	1.05	0.28	0.72	0.24	0.20
Ethyl acetate	Ethereal fruity, sweet	5	1.87	-	4.02	-	0.34	-	-	-	1.45	-	-	-	-	-	-	-	-	-	-
2,3-octanedione	Cilantro, herbal, earthy	12	-	-	-	-	-	-	-	-	2.64	-	-	-	-	-	-	-	-	-	-
β-ionone	Floral, woody, orris	0.007	2224.03	3552.98	2193.79	4570.20	1021.00	819.71	5010.53	2577.14	3245.05	2131.52	3296.83	2024.57	361.22	2311.03	3928.72	1768.57	-	-	1696.81
α-ionone	Woody, floral, fruity	2.6	-	1.85	-	-	0.74	-	-	-	-	-	-	-	-	-	0.64	1.34	-	-	-
Pentanoic acid	Sickening, putrid, acidic	0.36	-	-	-	-	-	-	-	-	-	-	3.53	-	-	-	-	-	-	-	-
2-methoxy-3-(2-methylpropyl)-pyrazine	Green pea, bell pepper	0.002	81713. 36	82228. 74	119244. 64	132677. 51	89511. 10	82742. 39	137804. 01	133650. 42	156204. 77	133971. 88	91835. 00	86000. 00	153515. 11	123353. 71	164461. 45	116601. 79	124849. 39	111876. 64	##### ##
2-methoxy-Phenol	Spice, vanilla, woody	2	62.05	-	-	-	-	-	-	-	-	-	0.59	-	-	-	-	-	-	-	-
2-pentyl-Furan	Green, earthy, beany	6	3.01	-	19.85	5.16	-	-	-	-	7.27	21.29	0.00	-	-	9.41	16.84	-	11.56	1.47	11.61
2-ethyl-Furan	Beany, cocoa, bready	2.3	14.06	27.47	15.06	4.06	10.98	24.64	11.81	19.34	23.89	4.15	0.00	8.18	10.35	8.06	30.17	15.43	3.34	6.46	6.17

Aroma characteristic referenced in http://www.thegoodscentscompany.com/. -, not detected. W1, Fengshouxianjiao; W2, Sujiao 8; W3, Qingan 7; W4, Sujiao 18; W5, Longjiao 2; W6, Longjiao 10; W7, Longjiao 11; W8, Huamei 105; W9, 37–124; W10, 3F*106; W11, Tianjiao 20; W12, Tianjiao 23; W13, Hangjiao 2; W14, Hangjiao 8; W15, NO.171; S1, NO.212; S2, NO.221; S3, Sujiao 9; S4, HJF42.

Supplementary Table 2 lists 14 VOCs with an OAV greater than 0.2 but less than 1. Among these, 3-carene was described as having a citrus, herbal, and pine-like flavor, being present in all tested varieties. These VOCs were limited by threshold concentration and did not play a major role in the formation of green pepper flavor. However, these volatile compounds can affect the aroma of pepper, contributing to background odor.

### 3.10. Correlation analysis of index

**FIGURE 7:**
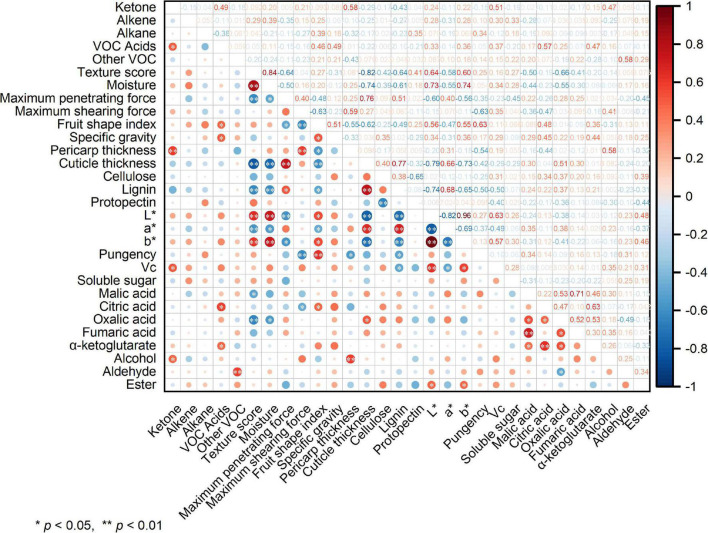
Heat map of the Pearson correlation analysis. Values are Pearson’s correlation coefficients. * and ^**^ denote correlation coefficients that are significant at the *p* < 0.05 and 0.01 level, respectively.

The correlation analysis in Figure 7 shows that the sensory score of pepper had an extremely significant negative correlation (*p* < 0.01) with the maximum penetrating force, cuticle thickness, lignin content, a* value, and oxalic acid content. Further, it exhibited a highly significant positive correlation with moisture content, L* value, and b* value, in addition to a significant negative correlation (*p* < 0.05) with malic acid content. Ketone content was positively correlated with those of alcohol, vitamin C, and VOC acids. Further, it exhibited an extremely significant correlation with pericarp thickness. The pepper L* value showed an extremely significant positive correlation with the b* value and vitamin C content, in addition to an extremely significant negative correlation with the a* value.

### 3.11. Pepper classification *via* principal component analysis

**FIGURE 8:**
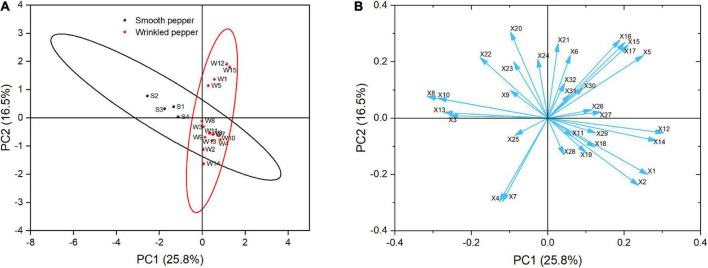
The PCA of pepper samples with different score **(A)** and loading **(B)** plot. Circles in panel **(A)** represent 95% Confidence Ellipse. The arrow in panel **(B)** indicates the loadings. X1: Texture score, X2: Moisture content, X3: Maximum penetrating force, X4: Maximum shearing force, X5: Fruit shape index, X6: Specific gravity, X7: Pericarp thickness, X8: Cuticle thickness, X9: Cellulose content, X10: Lignin, X11: Protopectin, X12: a* value, X13: b* value, X14: L* value, X15: Capsaicin, X16: Dihydrocapsaicin, X17: Pungency, X18: Vitamin C, X19: Soluble sugar, X20: Malic acid, X21: Citric acid, X22: Oxalic acid, X23: Fumaric acid, X24: α-ketoglutarate, X25: Alcohol, X26: Aldehyde, X27: Ester, X28: Ketone, X29: Alkene, X30: Alkane, X31: VOC Acid, X32: Other VOC.

It is difficult to evaluate the quality of fruit with only one index. Multivariate chemometric pattern recognition (PCA) was used to identify similarities in 32 indices among the evaluated pepper samples. As shown in Figure 8A, the first and second components (PC1 and PC2) explained 25.8 and 16.5% of the total variance, respectively. The values plotted in single ellipses were considered as confidence intervals. The wrinkled peppers can be distinguished from the smooth peppers along PC1, which contains mainly color and texture-related indices. The wrinkled peppers could be distinguished along PC2, which mainly represents the flavor- and texture- related indices. Loading analysis was used to identify the indices responsible for the distribution and differentiation of the samples in the current score plots, as shown in Figure 8B. The results showed that a*(X12) and L* (X14) had the largest positive contribution to PC1, while cuticle thickness (X8) and lignin content (X10) had the largest negative contribution. Malic acid (X20) and dihydrocapsaicin (X16) had the largest positive contribution to PC2, while maximum shear force (X4) and pericarp thickness (X7) had a negative contribution. The protopectin (X11) and VOC acid (X31) content had little effect on the differentiation of the pepper variety.

### 3.12. Calculation of the comprehensive membership function value

In fuzzy mathematics, the membership function method accumulates the membership value of each index of the evaluated variety and obtains a mean number. 50 indices were used in our calculations. Among these, indicators that adversely affect texture and acids, such as pentanoic acid, were regarded as negative indicators. In Table 3, larger mean values of the membership function indicate greater variety superiority. Our data showed that the comprehensive membership values of wrinkled peppers were higher than those of smooth pepper varieties.

**TABLE 3:** Membership function values of pepper quality index in 19 varieties.

Varieties	W1	W2	W3	W4	W5	W6	W7	W8	W9	W10	W11	W12	W13	W14	W15	S1	S2	S3	S4
Membership function value	0.461	0.528	0.516	0.556	0.418	0.396	0.431	0.438	0.505	0.371	0.378	0.359	0.473	0.471	0.575	0.333	0.242	0.269	0.327
Ranks	8	3	4	2	11	12	10	9	5	14	13	15	6	7	1	16	19	18	17

W1, Fengshouxianjiao; W2, Sujiao 8; W3, Qingan 7; W4, Sujiao 18; W5, Longjiao 2; W6, Longjiao 10; W7, Longjiao 11; W8, Huamei 105; W9, 37–124; W10, 3F*106; W11, Tianjiao 20; W12, Tianjiao 23; W13, Hangjiao 2; W14, Hangjiao 8; W15, NO.171; S1, NO.212; S2, NO.221; S3, Sujiao 9; S4, HJF42.

## 4. Discussion

### 4.1. Differences in texture and nutritional quality of pepper fruit

Among breeders, the most visible traits of new pepper varieties include fruit size, shape, color, and production-related traits (32). However, texture and flavor are often neglected. The present findings indicated that the 19 pepper varieties evaluated differed significantly not only in external shape, but also in internal texture. Differences in the cell wall structure and cuticle deposition between varieties could be important factors for fruit hardness, affecting storage and transportation resistance, and also having a negative impact on the consumer’s sensory experience of texture. Cellulose is the main structural component of plant cell walls, and its combination mode and significantly impact the texture of plant-derived foods. Lignin is a complex phenolic compound formed by further transportation and polymerization after the synthesis of lignin monomers through the phenylpropane metabolic pathway, and is important for enhancing rigidity to protect plants against pathogen attacks and mechanical stress (33). The maximum penetrating and shearing forces of pepper were estimated as sample firmness. Through sensory evaluation and physicochemical property analysis, it was shown that key indexes affecting the texture of pepper are cuticle thickness, maximum penetrating force, lignin content, and moisture content. In addition, correlation analysis showed that cuticle thickness was significantly positively correlated with the maximum penetrating force and lignin content, while negatively correlated with the sensory score of texture and fruit moisture content. Therefore, cuticle thickness is an important index that affects the texture quality of pepper fruits. The thin cuticle of wrinkled pepper was widely recognized by evaluators.

The color of fruits, which is an important criterion for consumers when purchasing vegetables, was determined based on their chlorophyll content at the green mature stage. Analysis of color differences showed that all pepper samples had negative values for a*, with positive values for L* and b*. The L* value indicated that smooth pepper fruits were darker than wrinkled pepper fruits, especially for variety NO.221. Fengshouxianjiao and 37–124 varieties show a brighter color. These characteristics indicate that most wrinkled peppers have better internal texture and external appearance. The color difference is significantly related to vitamin C content and fruit texture, including cuticle thickness, lignin content, and moisture content. However, whether there is a relationship between texture and color requires further study.

Recently, research has been conducted on the chemical composition of various pepper varieties (34, 35). Pungency is characterized by a pungent taste or a sensation of heat upon consumption. Capsaicin and dihydrocapsaicin account for approximately 90% of capsaicinoids and are therefore the most important components affecting pungency (36). There are reports that differences in capsaicinoid profiles within a given cultivar are controlled by a single dominant gene (37). Some studies have linked capsaicinoids in chili peppers to the plant’s defense mechanisms against certain pathogens, suggesting that pungency characteristics can be extremely variable and highly sensitive to plant growth conditions (38, 39). Therefore, uniform growth conditions were established for all 19 experimental varieties. Our results showed that the capsaicin and dihydrocapsaicin contents varied significantly within the germplasm of the 19 pepper varieties, which could be due to the genetic makeup of the pepper varieties used. Premium-wrinkled peppers are marketed in China under the quality label “Appetizing and Spicy.” The pungency of 15 wrinkled pepper varieties ranged from 1748.9 to 25529.4 SHU, which can be considered slightly pungent (700–3,000 SHU) to very pungent (25,000–70, 000 SHU), while the pungency of smooth varieties range from 711.45 to 8533.70, with slightly to moderately pungent (3,000–25,000 SHU) (40). Wrinkled peppers are a better choice for spicy lovers.

Pepper is a good source of nutrients and antioxidant compounds, especially vitamin C. Howard L. R. et al. determined the total ascorbic acid content of processed Jalapeños and fresh pepper (*C. annuum* L.) by high-performance liquid chromatography (HPLC) at the green and red maturity stages between 76.1 and 243.1 mg 100 g^–1^ (41). The recommended daily dose of vitamin C is 100 mg for adults (42). In this study, six varieties of peppers had higher vitamin C content. The consumption of 100 g of Fengshouxianjiao, Sujiao 8, Sujiao 18, 37–124, 3F*106, and HJF42 would meet the recommended daily intake of this essential nutrient. However, the same amounts of tomato and eggplant do not satisfy this requirement (43). The main soluble sugars in peppers are sucrose, glucose, and fructose. In general, soluble sugar and acid are the main flavor substances of non-spicy peppers. In this study, several varieties with low capsaicinoid content did not exhibit considerable soluble sugar content. Thus, soluble sugar is not the main metabolic substance causing the difference between pepper varieties. Five organic acids, including citric acid, malic acid, fumaric acid, α-ketoglutarate, and oxalic acid, were identified in the tested peppers and are well-perceived by human sour taste receptors. Citric acid was the dominant organic acid. Jarret et al. reported that the content of citric acid in 216 mature fruits of *Capsicum chinense* ranged from 2.44 to 8.18 mg⋅g^–1^ FW (29), which is consistent with our results. However, it is worth noting that oxalic acid is known to reduce calcium availability, which could increase the risk of kidney stones in humans (44). The average oxalic acid content of the smooth pepper was 26.19% higher than that of the wrinkled pepper. Therefore, in terms of human health, the high oxalic acid content in smooth pepper is a risk factor.

### 4.2. Aroma characteristics of pepper fruit

The aroma of pepper is one of its most important properties and represents a flavor sign for consumers. Cuevas-Glory et al. optimized HS-SPME for the detection and quantification of volatile compounds from habanero pepper *via* GC-MS and detected 53 compounds (45). In total, 127 compounds were previously identified in Chinese pepper (Paojiao) samples (46). In the present study, more than 190 volatile compounds were detected, confirming the diversity of volatiles in green pepper fruits. Studies have shown that different species, proportions, and the balance of volatile compounds in pepper fruits lead to differences in the taste of different pepper varieties (47). A study on Brazilian Malagueta chili peppers identified 83 compounds, with esters being the predominant class (13). Evaluation of volatile compounds in fresh pepper (C. *Chinense*) from Burundi identified 70 volatile compounds, with aliphatic esters, alcohols, terpenoids, and acids being the major classes (48). Discrepant and variable findings could be due to differences among pepper varieties. In this study, alcohols, aldehydes, and alkenes were the three most commonly detected volatile classes, whereas alcohols, aldehydes, and esters were the most abundant. The average volatile content of wrinkled pepper is 39.79% higher than that of smooth pepper, indicating that wrinkled pepper has superior odor characteristics.

El-Ghorab et al. reported that the major volatiles in fresh and dried pepper are benzaldehyde, 2-methoxy-3-isobutyl-pyrazine, and Z-β-ocimene (49). The major volatile compounds in Habanero chili peppers were hexyl isopentanoate, (Z)-3-hexenyl isopentanoate, hexyl pentanoate, and 3,3-dimethylcyclohexanol (47). In fresh and processed chili peppers and cachucha pepper, hexyl isopentanoate, hexyl pentanoate, hexyl 2-methylbutanoate, and 3,3-dimethylcyclohexanol were the major compounds (50). In the present study, 13 volatile compounds are common to all varieties. These included (E)-3-hexen-1-ol, *trans*-4-methylcyclohexanol, linalool, *trans*-2-hexenal, hexanal, heptanal, decanal, nonanal, (E, E)-2,4-decadienal, methyl salicylate, ethyl salicylate, 3-carene, and 2-isobutyl-3-methoxypyrazine.

Aldehydes have a major contribution to the formation of pepper odor, accounting for 48.3% of the 29 odor-active volatiles determined in this study. Aldehydes are important for a low odor threshold, which has been described for green leaves, cucumber, pungent, or herbaceous odor notes in Capsicum sniff analysis (51). Previous studies have shown that C6 and C9 aldehydes are the main sources of green aroma and are involved in fatty acid metabolism (52). Unsaturated fatty acids undergo stereospecific oxygenation to form 9-hydroperoxy and 13-hydroperoxy intermediates, which are further metabolized *via* the two lipoxygenase pathway branches to yield volatile compounds such as hexanal, (E)-3-hexenol, nonanal, and (E)-2-hexenal (53, 54). However, not all volatile compounds significantly affect pepper odor formation. Considering the odor threshold, *trans*-2-hexenal, hexanal, heptanal, nonanal, and (E,E)-2,4-decadienal were present in all varieties and may thus significantly contribute to pepper odor formation. Other volatile compounds, such as (E, E)-2,4-non-adienal, decanal, and 2-hexenal, only affect the odor formation of some pepper varieties. In addition, 2-isobutyl-3-methoxypyrazine is a flavor component that occurs naturally in green pepper, green peas, and asparagus, roughly defined as having a green pea, and green bell pepper odor (55). Previous studies have shown that the flavor of jalapeno pepper can be attributed to 2-isobutyl-3-methoxypyrazine (56). In this study, 2-isobutyl-3-methoxypyrazine contributed the most to the odor of 19 pepper varieties due to its extremely low threshold concentration (0.002 μg kg^–1^), thus playing an indispensable role in the green fragrance of peppers. Overall, the green odor could be representative of pepper fruit.

Ketones, including short-chain and methyl ketones, are also known as strong aroma compounds in Capsicum (odor notes of paprika and green pepper) (57). β-Ionone is a spice with a strong floral and woody odor. Although its content in tomatoes is very low, it contributes significantly to the formation of tomato odor (52). In this study, β-ionone was not found in two smooth-skinned peppers (Sujiao 9 and NO.221), but its OAV was much greater in all wrinkled peppers. In addition, the OAV of α-ionone in Sujiao 8 and NO.212 was greater than 1, indicating that it could be an aroma-active compound in these two varieties. Linalool is a natural monoterpenoid found in plants, such as coriander, and has been shown to have anti-injury, antibacterial, and anti-inflammatory activities (58). Linalool is a potent odorant of tea and imparts tea products with a creamy, floral odor, having a low threshold of perception (59). Although linalool has been detected in all pepper varieties, it does not appear to be the active odor component of Sujiao 9 and NO. 221, two smooth-skinned varieties, due to the limitation of OAV. In addition, undecanal and benzeneacetaldehyde can provide floral flavors for some pepper varieties.

Fruit flavor is another important characteristic of pepper. For example, 3-methyl-butanal is naturally present in essential oils such as citrus and lemon, having an apple-like aroma when highly diluted, similar to peach with low concentration. 1-Hexanol, β-cyclocitral, and ethyl acetate provide a sweet, fruity smell. Even nonanal, decanal, and (E, E)-2,4-decadienal have citrus and orange-like odors. Different combinations of green vegetable, floral, and fruity odor-contributing volatiles can explain the range of aroma sensations found in green peppers. Of course, the richness of pepper smell is not limited to these three flavor types. For instance, 1-octen-3-ol plays a role in the odor formation of eight varieties, with the attractive mushroom aroma, while 2-pentyl-furan contributes to the odor of earthy and beany, and 2-methoxy-phenol has a spicy, vanilla, and woody-like odor. However, there are some volatile compounds whose odor thresholds cannot be determined, and their importance in terms of contribution to the flavor of pepper remains unknown. Moreover, the characteristic flavor of pepper is formed by the combination of these compounds in a certain ratio, with the ratios determining unique flavors requiring further research.

In this study, the quality of 19 pepper varieties (*Capsicum annuum* L.) was characterized in detail at the green maturity stage. In addition to the significant differences in shape and color, assessment of firmness, nutrients, pungency, acids, and volatile compounds in different pepper types indicated that the quality of wrinkled pepper fruits differed from that of smooth pepper in terms of texture and flavor. Sensory evaluation showed that assessors preferred the texture of wrinkled pepper, which was mainly related to the cuticle thickness, lignin, and moisture content of the fruit. In addition, wrinkled pepper has a higher degree of pungency and a lower oxalic acid content than smooth pepper. The main groups of volatile compounds present in the green peppers were alcohols, aldehydes, and alkenes. Green odors and volatile compounds, such as 2-methoxy-3-(2-methylpropyl)-pyrazine, nonanal, (E)-2-octenal, hexanal, (E)-2-hexenal, (E, E)-2,4-heptadienal, (E)-3-hexen-1-ol, β-ionone, octanal, and 1-octen-3-ol, were mainly formed by the pepper aroma. Our findings characterize the properties of wrinkled peppers and provide theoretical evidence for the flavor description of pepper fruits. Nevertheless, additional investigation is required to minimize the undesirable texture of pepper and optimize flavor acceptance.

## Data availability statement

The original contributions presented in this study are included in the article/Supplementary material, further inquiries can be directed to the corresponding author.

## Author contributions

JX and JY conceived and designed the experiments. JZ, CW, YY, KH, and JinL conducted the experiments. JZ and JW analyzed the data and prepared the figures and illustrations. JZ wrote the manuscript. JZ, JX, JY and JiaL were involved in the related discussion. JX and EB helped to improve the quality of the manuscript. All authors have read and agreed to the published version of the manuscript.
