# Supplementary material for: Comprehensive fruit quality assessment and identification of aroma-active compounds in green pepper (Capsicum annuum L.)
Source: Front Nutr. 2023 Jan 10;9:1027605. doi: 10.3389/fnut.2022.1027605 (PMC9871545; doi:10.3389/fnut.2022.1027605)
Supplement: Supplementary file 1 [file Table_1.docx]

Supplementary Table1 The average weight of the fruits at the commodity maturity stage

| Varieties | Weight (g) | Varieties | Weight (g) |
| --- | --- | --- | --- |
| W1 | 29.6 ± 1.5 h | W11 | 72.44 ± 2.69 cde |
| W2 | 80.16 ± 3.03 bc | W12 | 26.37 ± 1.04 hi |
| W3 | 42.26 ± 1.65 g | W13 | 46.84 ± 1.22 fg |
| W4 | 73.86 ± 4.42 bcde | W14 | 90.52 ± 6.35 a |
| W5 | 54.02 ± 2.64 f | W15 | 12.7 ± 0.37 j |
| W6 | 75.82 ± 2.88 bcd | S1 | 22.33 ± 0.94 hi |
| W7 | 65.29 ± 4.02 e | S2 | 19.9 ± 1.25 ig |
| W8 | 68.66 ± 3.78 de | S3 | 41.49 ± 0.84 g |
| W9 | 82.14 ± 3.24 b | S4 | 45.68 ± 2.9 fg |
| W10 | 48.17 ± 2.15 fg |  |  |

Note: W1, Fengshouxianjiao; W2, Sujiao 8; W3, Qingan 7; W4, Sujiao 18; W5, Longjiao 2; W6, Longjiao 10; W7, Longjiao 11; W8, Huamei 105; W9, 37-124; W10, 3F*106; W11, Tianjiao 20; W12, Tianjiao 23; W13, Hangjiao 2; W14, Hangjiao 8; W15, NO.171; S1, NO.212; S2, NO.221; S3, Sujiao 9; S4, HJF42

Supplementary Table 2 Composition and content of volatile compounds in from 19 pepper varieties

| No.^a^ | CAS^b^ | Compounds^c^ | MF^d^ | MW^e^ | m/z^f^ | RT (min)^g^ | Contents (μg·kg^-1^)^h^ | | | | | | | | | | | | | | | | | | | |
| --- | --- | --- | --- | --- | --- | --- | --- | --- | --- | --- | --- | --- | --- | --- | --- | --- | --- | --- | --- | --- | --- | --- | --- | --- | --- | --- |
|  |  |  |  |  |  |  | W1 | W2 | W3 | W4 | W5 | W6 | W7 | W8 | W9 | W10 | W11 | W12 | W13 | W14 | W15 | S1 | S2 | S3 | S4 |  |
|  |  | Alcohols (55) |  |  |  |  |  |  |  |  |  |  |  |  |  |  |  |  |  |  |  |  |  |  |  |  |
| 1 | 4254-15-3 | (S)-(+)-1,2-Propanediol | C_3_H_8_O_2_ | 76.09 | 31.0, 43.1, 45.1 | 1.39 | 39.04 | 21.26 | - | - | - | 47.11 | - | 14.6 | - | - | - | - | - | - | - | - | - | - | - |  |
| 2 | 67-63-0 | Isopropyl Alcohol | C_3_H_8_O | 60.10 | 41.1, 45.1, 46.0 | 1.98 | - | - | - | - | - | - | - | - | - | - | - | - | - | - | - | 92.97 | - | - | - |  |
| 3 | 19132-06-0 | [S-(R*,R*)]-2,3-Butanediol | C_4_H_10_O_2_ | 90.12 | 43.8, 45.1, 75.0 | 2.22 | - | 11.2 | 3.97 | 16.6 | - | 3.53 | 4.27 | 4.07 | 4.17 | 3.52 | 2.69 | 3.76 | - | 4.6 | 14.02 | 0.24 | 3.26 | - | 6.5 |  |
| 4 | 513-85-9 | 2,3-Butanediol | C_4_H_10_O_2_ | 90.12 | 44.1, 45.1, 75.0 | 2.22 | - | 14.49 | - | - | - | - | - | - | - | - | - | - | - | - | - | - | - | 1.13 | - |  |
| 5 | 111-46-6 | Diethylene glycol | C_4_H_10_O_3_ | 106.12 | 43.1, 45.1, 74.9 | 2.24 | - | - | - | - | - | 3.08 | - | - | - | - | 5.43 | 5.36 | - | 4.61 | 5.78 | - | - | 3.04 | 3.65 |  |
| 6 | 24347-58-8 | (2R,3R)-(-)-2,3-Butanediol | C_4_H_10_O_2_ | 90.12 | 43.1, 45.1, 75.0 | 2.32 | - | - | - | - | - | - | - | 1.09 | - | - | - | - | - | - | - | - | - | - | - |  |
| 7 | 24621-61-2 | (S)-1,3-Butanediol | C_4_H_10_O_2_ | 90.12 | 43.1, 45.0, 57.0 | 2.33 | - | - | - | - | 19.48 | - | - | - | 0.11 | - | - | - | - | - | - | - | - | - | - |  |
| 8 | 928-40-5 | 1,5-Hexanediol | C_6_H_14_O_2_ | 118.17 | 43.1, 56.1, 75.0 | 2.40 | - | - | - | - | - | - | - | - | 0.48 | - | - | - | - | - | - | - | - | - | - |  |
| 9 | 625-31-0 | 4-Penten-2-ol | C_5_H_10_O | 86.13 | 39.0, 42.0, 45.0 | 2.44 | - | - | - | - | - | - | - | - | - | - | 0.14 | - | - | - | - | - | - | - | - |  |
| 10 | 4435-50-1 | 1,2,3-Butanetriol | C_4_H_10_O_3_ | 106.12 | 43.1, 57.0, 75.0 | 2.72 | - | 0.75 | - | - | - | 0.39 | - | - | - | - | - | - | - | - | - | - | 0.52 | - | - |  |
| 11 | 50551-88-7 | 5-Methylhex-5-en-2-ol | C_7_H_14_O | 114.19 | 43.1, 55.1, 57.1 | 2.92 | - | - | - | - | - | - | - | - | 0.24 | - | - | - | - | - | - | - | - | - | - |  |
| 12 | 19550-05-1 | 3,4-Dimethyl-2-Hexanol | C_8_H_18_O | 130.23 | 44.0, 57.1, 78.1 | 3.03 | - | - | - | - | - | - | - | 0.42 | 1.91 | - | - | - | - | - | - | - | - | - | - |  |
| 13 | 96-41-3 | Cyclopentanol | C_5_H_10_O | 86.13 | 45.0, 57.0, 74.9 | 3.14 | - | - | - | - | - | - | - | - | - | 1.27 | - | - | - | - | - | - | - | - | - |  |
| 14 | 16326-97-9 | cis-1,3-Cyclopentanediol | C_5_H_10_O_2_ | 102.13 | 43.1, 55.0, 57.0 | 3.20 | - | - | 0.72 | - | 0.08 | - | - | 1.38 | - | - | - | - | - | - | - | 4.12 | - | - | - |  |
| 15 | 31367-46-1 | 3-Methylheptan-2-ol | C_8_H_18_O | 130.23 | 44.7, 45.1, 57.0 | 3.22 | - | - | - | - | - | - | - | - | - | - | - | - | 0.21 | - | - | - | - | - | 0.48 |  |
| 16 | 7731-29-5 | trans-4-Methylcyclohexanol | C_7_H_14_O | 114.19 | 55.0, 81.0, 96.0 | 3.42 | 15.46 | 2.01 | 16.48 | 0.78 | 7.6 | 24.1 | 14.29 | 4.08 | 3.1 | 5.42 | 16.48 | 1.34 | 6.09 | 4.56 | 9.12 | 10.94 | 5.36 | 5.08 | 2.21 |  |
| 17 | 7383-19-9 | 1-Heptyn-3-ol | C_7_H_12_O | 112.17 | 42.1, 55.0, 57.3 | 4.35 | - | 0.65 | 0.16 | - | - | - | - | - | - | - | - | - | - | - | 0.46 | - | - | - | - |  |
| 18 | 1576-95-0 | (Z)-2-Penten-1-ol | C_5_H_10_O | 86.13 | 55.0, 57.0, 70.0 | 5.10 | - | - | - | - | 5.17 | - | - | - | - | - | - | - | - | - | - | - | - | - | - |  |
| 19 | 51174-44-8 | 3-Methylpent-4-en-1-ol | C_6_H_12_O | 100.16 | 41.1, 55.0, 67.0 | 7.35 | 8.82 | 15.8 | - | - | - | - | - | - | - | - | - | - | - | - | - | - | - | - | - |  |
| 20 | 1572-08-3 | 3-Methylpenta-2,4-dien-1-ol | C_6_H_10_O | 98.14 | 70.0, 83.0, 93.0 | 7.37 | - | 1069.92 | 747.47 | 380.7 | 330.65 | - | 231.82 | 665.99 | 709.61 | - |  | 138.9 | - | 269.98 | - | 221.19 | 290.61 | 342.41 | - |  |
| 21 | 822-67-3 | 2-Cyclohexen-1-ol | C_6_H_10_O | 98.14 | 70.0, 83.0, 98.0 | 7.39 | - | 1045.17 | - | - | 894.62 | - | 627.41 | - | 925.68 | 627.53 | 634.87 | 473.78 | 272.78 | - | 945.55 | 350.63 | - | - | 333.32 |  |
| 22 | 928-97-2 | (E)-3-Hexen-1-ol | C_6_H_12_O | 100.16 | 41.1, 67.0, 82.0 | 7.61 | 390.06 | 377.99 | 200.27 | 414.08 | 491.99 | 417.04 | 425.8 | 659.08 | 361.89 | 274.44 | 699.3 | 627.9 | 414.25 | 737.43 | 363.83 | 326.28 | 347.29 | 528.52 | 379.69 |  |
| 23 | 928-92-7 | (E)-4-Hexen-1-ol | C_6_H_12_O | 100.16 | 39.0, 67.0, 82.0 | 7.62 | 295.91 | 172.93 | - | - | 114 | - | - | 186.61 | - | - |  | 143.98 | - | - | - | - | 214.82 | - | - |  |
| 24 | 20461-31-8 | 2-Methylene cyclopentanol | C_6_H_10_O | 98.14 | 69.0, 83.0, 98.0 | 7.77 | - | 627.39 | 1131.7 | - | - | 307.88 | - | 933.23 | - | 150.6 | 230 | 310 | 326.37 | 286.79 | - | - | 336.73 | 255.11 | 492.38 |  |
| 25 | 928-94-9 | (Z)-2-Hexen-1-ol | C_6_H_12_O | 100.16 | 41.1, 56.1, 67.0 | 7.85 | - | - | - | - | 212.82 | - | - | - | - | - | - | - | - | - | - | - | - | - | - |  |
| 26 | 42072-39-9 | (S)-(+)-3-Methyl-1-pentanol | C_6_H_14_O | 102.18 | 43.1, 56.1, 69.0 | 7.90 | 834.42 | 199.79 | 491.55 | 532.54 | 591.59 | 407.68 | 499.89 | - | 407.79 | 590.01 | 324.76 | 300.13 | 550.71 | 284.25 | 506.55 | 520.68 | 751.05 | 333.13 | 419.87 |  |
| 27 | 111-27-3 | 1-Hexanol | C_6_H_14_O | 102.18 | 56.1, 57.0, 69.0 | 7.95 | 140.68 | 235.7 | 489.34 | - | 427.64 | 474.29 | - | 462.72 | 311.91 | 134.91 | 244.19 | 93.3 | 307.07 | 276.06 | - | 239.94 | - | - | 264.09 |  |
| 28 | 31502-14-4 | (E)-2-Nonen-1-ol | C_9_H_18_O | 142.24 | 41.1, 43.1, 55.0 | 8.73 | 0.67 | - | - | - | - | - | - | - | - | - | - | - | - | - | - | - | 7.6 | - | - |  |
| 29 | 4065-80-9 | 2-Methylenecyclohexanol | C_7_H_12_O | 112.17 | 41.1, 69.9, 83.0 | 10.40 | - | 27.56 | - | 6.64 | 10.82 | 20.58 | - | - | 58.42 | - | - | 21.55 | 36.6 | - | 18.83 | - | - | 13.04 | - |  |
| 30 | 928-96-1 | cis-3-Hexen-1-ol | C_6_H_12_O | 100.16 | 67.0, 82.0, 83.0 | 10.59 | - | - | - | - | - | - | - | - | - | - | 2.67 | - | - | - | - | - | - | - | - |  |
| 31 | 818-72-4 | 1-Octyn-3-ol | C_8_H_14_O | 126.20 | 43.1, 55.0, 70.0 | 10.64 | - | 0.57 | 2.85 | - | - | - | - | - | - | - | 0.14 | - | - | - | - | - | - | - | - |  |
| 32 | 21078-65-9 | 2-Ethyl-1-decanol | C_12_H_26_O | 186.33 | 55.0, 57.0, 96.9 | 11.07 | - | - | - | - | 8.09 | - | - | - | 3.02 | - | - | - | - | - | - | - | - | - | - |  |
| 33 | 75039-84-8 | trans-2-Undecen-1-ol | C_11_H_22_O | 170.29 | 43.1, 57.0, 82.0 | 11.10 | - | - | 1.49 | - | - | 1.96 | 1.35 | - | - | - | - | 1.85 | 1.14 | - | - | - | 0.74 | - | 3.13 |  |
| 34 | 30385-19-4 | 1,7-Octadien-3-ol | C_8_H_14_O | 126.20 | 43.1, 57.0, 70.0 | 11.10 | - | - | - | - | - | - | - | - | - | - | - | - | - | - | - | - | - | - | 5.55 |  |
| 35 | 3391-86-4 | 1-Octen-3-ol | C_8_H_16_O | 128.21 | 43.1, 57.0, 72.0 | 11.10 | 2.05 | 2.89 | 7.17 | - | 8.16 | - | - | - | 1.08 | 8.5 |  | - | - | - | 8.21 | - | - | 5.21 | - |  |
| 36 | 18680-27-8 | (1S,2S,3R,5S)-(+)-Pinanediol | C_10_H_18_O_2_ | 170.25 | 43.1, 99.0, 126.0 | 11.30 | - | - | - | - | - | - | - | - | 22.17 | - | - | - | - | 18.98 | 19.3 | - | - | - | - |  |
| 37 | 619-01-2 | 2-Methyl-5-(1-methylethenyl)-Cyclohexanol | C_10_H_18_O | 154.25 | 45.0, 83.0, 93.0 | 12.47 | - | - | - | - | 0.25 | - | - | - | - | - | - | - | - | - | - | - | - | - | - |  |
| 38 | 41453-56-9 | (Z)-2-nonen-1-ol | C_9_H_18_O | 142.24 | 43.1, 57.0, 83.0 | 12.51 | - | - | - | - | - | - | - | - | - | - | - | 50.13 | - | - | - | - | - | - | - |  |
| 39 | 27779-29-9 | (+)-isopinocampheol | C_10_H_18_O | 154.25 | 41.1, 55.1, 84.0 | 13.29 | - | - | - | - | - | - | 42.23 | - | 0.16 | - | 31.66 | 19.76 | - | - | - | 11.12 | - | - | 62.88 |  |
| 40 | 473-61-0 | 2,6,6-Trimethylbicyclo[3.1.1]heptan-3-ol | C_10_H_18_O | 125.03 | 69.0, 70.0, 83.0 | 13.83 | - | - | - | 22.7 | - | 54.23 | - | - | - | - | - | - | - | - | - | - | - | - | - |  |
| 41 | 99-48-9 | Carveol | C_10_H_16_O | 152.23 | 55.1, 91.0, 108.8 | 14.20 | - | - | - | 6.5 | 3.53 | - | 0.18 | - | 4.31 | 18.46 | - | 2.87 | - | - | - | - | - | - | 12.01 |  |
| 42 | 5978-70-1 | (R)-2-Octanol | C_8_H_18_O | 130.23 | 45.1, 55.0, 83.0 | 14.24 | 513.75 | - | - | - | - | - | - | - | - | - | 48.31 | - | - | - | - | - | - | - | - |  |
| 43 | 22422-34-0 | (1R,2R,3S,5R)-(-)-2,3-Pinanediol | C_10_H_18_O_2_ | 170.25 | 43.1, 55.0, 83.0 | 14.29 | - | 21.98 | - | - | 32.55 | - | - | - | 12.97 | - |  | - | 32.01 | - | - | - | - | - | - |  |
| 44 | 78-70-6 | Linalool | C_10_H_18_O | 154.25 | 57.0, 71.0, 92.9 | 14.41 | 9.58 | 24.28 | 17 | 10.27 | 30.63 | 38.44 | 23.35 | 17.86 | 36.65 | 39.7 | 23.2 | 14.74 | 23.72 | 23.7 | 59.83 | 3.22 | 7.3 | 4.85 | 14.15 |  |
| 45 | 19894-97-4 | (-)-Myrtenol | C_10_H_16_O | 152.23 | 43.1, 91.0, 118.9 | 19.64 | - | - | - | - | - | - | - | - | - | - | - | - | - | - | - | - | 0.85 | - | - |  |
| 46 | 55722-60-6 | cis-Chrysanthenol | C_10_H_16_O | 152.23 | 79.0, 80.9, 120.9 | 19.77 | - | - | - | - | - | - | - | - | - | - | - | - | - | - | 8.51 | - | 3 | - | - |  |
| 47 | 112-72-1 | 1-Tetradecanol | C_14_H_30_O | 214.39 | 43.1, 57.1, 83.0 | 21.35 | - | - | - | - | 10.82 | - | - | - | - | - | - | - | - | - | - | - | - | 4.52 | - |  |
| 48 | 23445-02-5 | Cubebol | C_15_H_26_O | 222.37 | 92.7, 121.0, 206.6 | 21.84 | - | - | - | 5.06 | 4.35 | 1.18 | - | - | - | - | - | - | 2.48 | - | - | - | - | 2.55 | 3.25 |  |
| 49 | 58319-05-4 | (Z)-sesquisabinene hydrate | C_15_H_26_O | 222.37 | 80.7, 118.9, 206.9 | 23.07 | - | 0.91 | - | 0.6 | 1.02 | 0.19 | 0.69 | 2.22 | 2.14 | - | - | 1.87 | - | - | 17.33 | - | - | - | - |  |
| 50 | 7212-44-4 | Nerolidol | C_15_H_26_O | 222.37 | 69.0, 92.9, 106.9 | 24.95 | - | 2.04 | - | - | - | - | - | - | - | - | - | - | - | - | - | - | - | - | - |  |
| 51 | 40716-66-3 | (±)-trans-Nerolidol | C_15_H_26_O | 222.37 | 69.1, 92.9, 206.8 | 24.95 | - | 6.53 | - | 2.32 | 1.48 | - | - | 4.93 | 3.03 | - | 2.88 | - | - | - | - | 7.8 | - | - | 3.53 |  |
| 52 | 1000285-43-5 | Nerolidol 1 | C_15_H_26_O | 222.37 | 79.0, 93.0, 106.9 | 24.95 | - | - | - | - | - | - | - | - | 2.92 | - | - | - | - | - | - | - | - | - | - |  |
| 53 | 1724-39-6 | Cyclododecanol | C_12_H_24_O | 184.32 | 67.0, 69.0, 82.1 | 25.15 | - | 8.96 | - | - | - | - | - | - | - | 2.2 | - | - | - | - | - | - | - | - | - |  |
| 54 | 30689-78-2 | (R)-(-)-(Z)-14-Methyl-8-hexadecen-1-ol | C_17_H_34_O | 254.45 | 44.0, 57.0, 82.0 | 25.82 | 4.43 | 1.49 | - | - | 3.22 | - | - | 2.71 | - | - | 14.53 | - | - | - | 2.6 | - | - | - | - |  |
| 55 | 4727-17-7 | Cyclopentadecanol | C_15_H_30_O | 226.40 | 68.0, 94.9, 95.9 | 27.77 | - | - | - | - | - | - | 4.14 | - | - | - | - | - | - | - | - | - | - | - | - |  |
|  |  | Aldehydes (30) |  |  |  |  |  |  |  |  |  |  |  |  |  |  |  |  |  |  |  |  |  |  |  |  |
| 56 | 109-87-5 | Methylal | C_3_H_8_O_2_ | 76.09 | 45.1, 75.0, 77.0 | 2.12 | - | - | - | - | - | 13.3 | - | - | - | - | 10.41 | 6.87 | - | - | - | - | - | - | - |  |
| 57 | 107-89-1 | 3-Hydroxy-butanal | C_4_H_8_O_2_ | 88.11 | 42.1, 44.1, 61.0 | 2.85 | - | - | - | - | - | - | - | - | - | - | - | - | 1.28 | - | 0.58 | - | - | - | - |  |
| 58 | 590-86-3 | 3-Methyl-butanal | C_5_H_10_O | 86.13 | 43.1, 45.1, 58.0 | 2.92 | - | - | - | - | - | - | - | - | - | - | - | - | - | - | 1.94 | - | 1.51 | - | - |  |
| 59 | 105-57-7 | 1,1-Diethoxyethane | C_6_H_14_O_2_ | 118.17 | 45.1, 73.0, 103.0 | 4.10 | 3.59 | 16.63 | 9.73 | 2.96 | 1.05 | 17.7 | 3.75 | 14.57 | - | 4.07 | 6.04 | 3.08 | 8.52 | 9.81 | 0.66 | 35.88 | 22.09 | 13.93 | 5.66 |  |
| 60 | 66-25-1 | Hexanal | C_6_H_12_O | 100.16 | 44.1, 72.0, 82.0 | 5.88 | 303.09 | 1163.92 | 1937.92 | 1394.77 | 915.08 | 961.04 | 917.67 | 1368.99 | 1303.57 | 1345.33 | 1146.19 | 1006 | 962.63 | 908.12 | 2168.94 | 617.54 | 466.83 | 253.39 | 642.27 |  |
| 61 | 111-71-7 | Heptanal | C_7_H_14_O | 114.19 | 44.1, 55.0, 70.0 | 6.44 | 12.72 | 9.9 | 9.7 | 5.39 | 11.45 | 15.35 | 1.43 | 11.83 | 9.29 | 14.52 | 2.48 | 0.74 | 12.09 | 0.19 | 14.26 | 2.23 | 6.19 | 2.02 | 2.61 |  |
| 62 | 6728-26-3 | Trans-2-Hexenal | C_6_H_10_O | 98.14 | 55.0, 67.0, 83.0 | 7.75 | 459.59 | 492.19 | 619.3 | 776.24 | 462.99 | 596.99 | 387.58 | 465.47 | 334.77 | 565.3 | 250.75 | 290.34 | 661.48 | 699.43 | 706.39 | 665.48 | 163.06 | 627.05 | 859.57 |  |
| 63 | 142-83-6 | (E,E)-2,4-Hexadienal | C_6_H_8_O | 96.13 | 41.1, 67.0, 80.9 | 9.10 | - | - | - | - | - | - | - | - | - | - | - | - | 13.69 | - | - | - | - | - | - |  |
| 64 | 505-57-7 | 2-Hexenal | C_6_H_10_O | 98.14 | 57.0, 69.0, 83.0 | 10.30 | 312.97 | - | - | - | - | - | - | - | - | - | 10.23 | - | - | - | - | - | - | - | - |  |
| 65 | 2043-61-0 | Cyclohexanecarboxaldehyde | C_7_H_12_O | 112.17 | 55.1, 70.0, 83.0 | 10.41 | - | - | 5.2 | - | - | - | - | - | - | - | - | - | - | - | 49.06 | - | - | - | 20.64 |  |
| 66 | 100-52-7 | Benzaldehyde | C_7_H_6_O | 106.12 | 77.0, 83.0, 104.9 | 10.55 | - | - | - | - | - | - | - | - | - | - | - | - | - | - | - | - | 7.61 | - | - |  |
| 67 | 112-45-8 | 10-Undecenal | C_11_H_20_O | 168.28 | 41.1, 57.0, 83.0 | 12.72 | - | - | - | - | - | 13.87 | - | - | 0.52 | - | 22.87 | 22.87 | - | - | - | - | - | - | - |  |
| 68 | 122-78-1 | Benzeneacetaldehyde | C_8_H_8_O | 120.15 | 65.0, 90.9, 91.9 | 12.91 | 12.12 | 7.96 | - | 3.07 | - | - | - | - | - | 3.15 | 6.69 | 4.29 | - | - | 17.29 | 3.79 | 8.73 | 6.52 | 3.06 |  |
| 69 | 2548-87-0 | (E)-2-Octenal | C_8_H_14_O | 126.20 | 57.0, 70.0, 83.0 | 13.28 | 52.14 | 34.24 | 58.29 | - | 81.14 | - | 43.15 | - | 60.82 | - | 14.79 | 8.77 | 56.45 | 30.06 | - | 30.86 | 16.25 | 31.04 | 28.46 |  |
| 70 | 18829-55-5 | (E)-2-Heptenal | C_7_H_12_O | 112.17 | 41.0, 55.0, 83.0 | 13.33 | - | - | - | - | - | - | - | - | - | - | 0.19 | - | - | - | - | - | - | - | - |  |
| 71 | 21391-98-0 | Phellandral | C_10_H_16_O | 152.23 | 71.0, 81.0, 108.9 | 14.04 | - | - | - | - | 0.21 | - | - | - | - | 2.27 | - | - | - | - | - | - | - | - | - |  |
| 72 | 5910-87-2 | (2E,4E)-2,4-Nonadienal | C_9_H_14_O | 138.21 | 43.1, 81.0, 82.0 | 14.37 | - | 42.03 | 7.6 | - | - | - | - | - | - | - | - | - | - | - | - | - | - | - | - |  |
| 73 | 124-19-6 | Nonanal | C_9_H_18_O | 142.24 | 57.0, 81.0, 98.0 | 14.52 | 30.84 | 37.81 | 22.04 | 10.25 | 21.82 | 30.74 | 14.46 | 18.05 | 30.37 | 19.2 | 17.57 | 44.45 | 30.84 | 27.77 | 27.63 | 17.77 | 15.52 | 14.87 | 27.69 |  |
| 74 | 18829-56-6 | (E)-2-Nonenal | C_9_H_16_O | 140.22 | 55.0, 70.0, 80.9 | 15.27 | 68.54 | - | - | - | 52.07 | - | 93.72 | 89.25 | - | - | 1.87 | - | 15.03 | - | - | - | - | 2.13 | - |  |
| 75 | 4748-78-1 | 4-Ethylbenzaldehyde | C_9_H_10_O | 134.18 | 90.9, 105.0, 118.9 | 16.06 | - | - | - | - | 10.38 | - | - | - | 5.5 | - | - | - | - | - | - | - | - | - | - |  |
| 76 | 34246-54-3 | 3-Ethyl-benzaldehyde | C_9_H_10_O | 134.18 | 90.9, 103.0, 132.9 | 16.07 | - | - | - | - | - | - | 3.51 | - | - | - | - | - | - | - | - | - | - | - | - |  |
| 77 | 112-31-2 | Decanal | C_10_H_20_O | 156.27 | 56.0, 80.9, 94.9 | 16.92 | 28.32 | 52.23 | 32.16 | - | 24.59 | 22.83 | 23.33 | 27.43 | 40.7 | 26.31 | 29.68 | 42.83 | 37.27 | 31.65 | 45.53 | 45.61 | 21.56 | 16.8 | 24.79 |  |
| 78 | 432-25-7 | β-Cyclocitral | C_10_H_16_O | 152.23 | 81.0 109.0, 136.9 | 17.49 | 8.27 | 5.5 | 4.7 | 2.38 | 2.78 | - | 3.54 | 3.63 | 3.99 | 3.02 | 2.16 | 1.3 | 3.37 | 3.56 | 5.52 | 3.03 | 1.99 | 1.63 | 1.68 |  |
| 79 | 25152-84-5 | (E,E)-2,4-Decadienal | C_10_H_16_O | 152.23 | 67.0, 79.0, 81.0 | 19.30 | 13.78 | 13.15 | 82.98 | 24.13 | 17.22 | 22.38 | 33.28 | 17.76 | 38.59 | 17.24 | 25.54 | 19.76 | 41.23 | 31.2 | 30.52 | 24.09 | 8.2 | 7.45 | 6.84 |  |
| 80 | 112-44-7 | Undecanal | C_11_H_22_O | 170.29 | 43.1, 56.9, 81.9 | 19.49 | 5.48 | 3.87 | 2.74 | - | - | 0.65 | - | - | - | - | - | - | - | - | - | - | - | 2.54 | - |  |
| 81 | 22644-96-8 | (E)-2-Hexadecenal | C_16_H_30_O | 238.41 | 43.1, 83.1, 111.0 | 21.35 | - | - | - | - | - | - | - | - | - | 3.7 | - | - | - | - | - | - | - | - | - |  |
| 82 | 10486-19-8 | Tridecanal | C_13_H_26_O | 198.35 | 43.1, 57.1, 82.0 | 23.78 | 73.75 | - | - | - | 6.9 | 3.7 | - | - | - | - | - | 3.58 | - | 8.2 | - | - | - | 18.88 | - |  |
| 83 | 51766-65-5 | .gamma.-Dehydro-ar-himachalene | C_15_H_20_ | 200.32 | 169.8, 170.9, 184.9 | 24.34 | - | 4.58 | - | 2.16 | - | - | 1.99 | 6.5 | 2.24 | 1.7 | 1.93 | - | - | - | - | - | - | - | - |  |
| 84 | 124-25-4 | Tetradecanal | C_14_H_28_O | 212.37 | 57.1, 82.0, 95.0 | 25.51 | 22.56 | 4.48 | - | - | 6.39 | - | - | 7.2 | - | 4 | 16.59 | 12.76 | - | - | 11.57 | 8.19 | - | 9.46 | - |  |
| 85 | 2765-11-9 | Pentadecanal | C_15_H_30_O | 226.40 | 67.0, 82.0, 96.0 | 27.73 | 122.57 | 40.36 | 8.51 | 4.09 | 67.1 | 18.71 | 9.46 | 29 | 11.24 | 41.25 | 51.07 | 57.6 | 23.28 | 25.53 | 54.31 | 33.8 | - | 123.38 | 17.13 |  |
|  |  | Esters (22) |  |  |  |  |  |  |  |  |  |  |  |  |  |  |  |  |  |  |  |  |  |  |  |  |
| 86 | 141-78-6 | Ethyl Acetate | C_4_H_8_O_2_ | 88.11 | 43.1, 70.1, 82.9 | 2.26 | 9.35 | - | 20.1 | - | 1.72 | - | - | - | 7.27 | - | - | - | - | - | - | - | - | - | - |  |
| 87 | 97-64-3 | Ethyl lactate | C_5_H_10_O_3_ | 118.13 | 44.2, 45.1, 75.0 | 2.28 | - | - | 14.09 | - | 8.08 | - | 2.32 | 8.01 | 1.37 | - | 10.05 | - | - | - | - | 1.69 | 10.61 | 3.82 | - |  |
| 88 | 22597-23-5 | 4-Methylcyclohexanol acetate | C_9_H_16_O_2_ | 156.22 | 57.0, 81.0, 96.0 | 3.65 | - | - | 2.7 | - | - | - | - | - | - | - | - | - | - | - | - | - | - | - | - |  |
| 89 | 61444-38-0 | cis-3-Hexenyl cis-3-hexenoate | C_12_H_20_O_2_ | 196.29 | 41.1, 67.0, 98.0 | 7.46 | 384.2 | 590.85 | - | 572.1 | 664.79 | 398.01 | 603.7 | 482.32 | 950.27 | 632.89 | 270.08 | 230.8 | 631.8 | 222.12 | 692.99 | 586.01 | 458.27 | 345.13 | 492.93 |  |
| 90 | 1000373-83-8 | Ethyl (E)-hex-3-enyl carbonate | C_9_H_16_O_3_ | 172.22 | 41.1, 67.0, 83.0 | 7.51 | 332.14 | - | - | - | - | 773.12 | - | - | - | 529.27 | 188.94 | 203.06 | - | - | - | - | - | - | - |  |
| 91 | 629-33-4 | Formic acid, heptyl ester | C_7_H_14_O_2_ | 130.19 | 43.1, 56.1, 67.0 | 7.94 | - | - | 580.69 | 359.84 | 225.67 | 389.1 | 174.47 | - | 64.95 | 297.88 |  | 74 | 521.52 | - | 277.8 | - | 110.44 | - | 217.81 |  |
| 92 | 112-23-2 | Hexyl formate | C_8_H_16_O_2_ | 144.21 | 43.1, 56.1, 70.1 | 10.91 | - | - | - | - | - | - | - | - | - | - | 4.73 | - | - | - | - | - | - | - | - |  |
| 93 | 106-70-7 | Hexanoic acid, methyl ester | C_7_H_14_O_2_ | 130.19 | 43.0, 74.0, 87.0 | 11.12 | - | - | - | - | - | - | - | - | - | - | 0.34 | - | - | - | - | - | - | - | - |  |
| 94 | 3050-69-9 | n-Caproic acid vinyl ester | C_8_H_14_O_2_ | 142.20 | 41.6, 43.1, 99.0 | 11.23 | 12.17 | - | 2.24 | - | 42.25 | - | 36.89 | 10.48 | 9.47 | 20.43 | - | 22.76 | 9.77 | 1.08 | 27.14 | 11.75 | 25.27 | - | 3.14 |  |
| 95 | 123-66-0 | Hexanoic acid, ethyl ester | C_8_H_16_O_2_ | 144.21 | 43.0, 73.0, 88.0 | 13.20 | - | - | - | - | - | - | - | - | - | - | 0.28 | - | - | - | - | - | - | - | - |  |
| 96 | 41519-23-7 | cis-3-Hexenyl iso-butyrate | C_10_H_18_O_2_ | 170.25 | 43.1, 67.0, 82.0 | 15.54 | - | - | - | - | - | - | - | - | - | - | - | - | - | - | 19.14 | - | - | - | - |  |
| 97 | 53398-84-8 | (E)-3-hexen-1-yl butyrate | C_10_H_18_O_2_ | 170.25 | 43.1, 67.0, 120.9 | 15.55 | - | - | - | - | - | - | - | - | - | - | - | 22.15 | - | - | 37.11 | - | - | - | - |  |
| 98 | 93-89-0 | Ethyl benzoate | C_9_H_10_O_2_ | 150.17 | 76.9, 104.9, 121.9 | 16.26 | - | 19.66 | - | 10.61 | - | - | 27.01 | 20.47 | 23.39 | - | - | 17.59 | - | - | - | 11.52 | - | - | - |  |
| 99 | 10482-77-6 | Citronellyl benzoate | C_17_H_24_O_2_ | 260.37 | 71.0, 89.0, 104.9 | 16.30 | - | - | - | 9.69 | - | - | - | - | - | - | - | - | - | - | - | - | - | - | - |  |
| 100 | 119-36-8 | Methyl salicylate | C_8_H_8_O_3_ | 152.15 | 91.9, 119.9, 120.9 | 16.90 | 76.97 | 117.15 | 88.37 | 109.57 | 119.37 | 66.12 | 103.98 | 100.92 | 98.67 | 126.89 | 42.88 | 42.35 | 61.93 | 97.77 | 200.26 | 156.45 | 167.23 | 88.98 | 75.89 |  |
| 101 | 7786-58-5 | Butanoic acid, 3-methyl-, octyl ester | C_13_H_26_O_2_ | 214.34 | 56.1, 84.0, 102.9 | 17.09 | - | - | - | 19.18 | - | - | 28.3 | - | - | - | 19.64 | 25.76 | - | - | 36.11 | 33.3 | - | - | 25.74 |  |
| 102 | 118-61-6 | Ethyl salicylate | C_9_H_10_O_3_ | 166.17 | 91.9, 119.9 165.9 | 18.74 | 16.27 | 27.63 | 21.58 | 27.13 | 27.18 | 28.05 | 37.54 | 33.47 | 27.38 | 37.01 | 19.9 | 16.63 | 29.74 | 28.51 | 88.02 | 23.44 | 60.6 | 20.14 | 17.04 |  |
| 103 | 35852-42-7 | 4-Methylpentyl 4-methylpentanoate | C_12_H_24_O_2_ | 200.32 | 56.0, 84.0, 117.0 | 19.64 | - | 10.49 | - | 26.12 | 5.18 | - | 16.2 | 24.12 | 11.61 | 9.65 | 6.2 | 6.88 | - | 3.33 | 24.85 | 18.06 | - | - | 14.24 |  |
| 104 | 76649-16-6 | Ethyl trans-4-decenoate | C_12_H_22_O_2_ | 198.30 | 69.0, 96.9, 136.9 | 20.29 | - | - | - | - | - | - | - | - | - | - | - | - | - | - | 19.73 | - | - | - | - |  |
| 105 | 110-38-3 | Decanoic acid, ethyl ester | C_12_H_24_O_2_ | 200.32 | 43.1, 88.0, 156.9 | 20.65 | - | - | - | - | - | - | - | - | - | - | - | - | - | - | 8.89 | 8.16 | - | - | - |  |
| 106 | 106-33-2 | Dodecanoic acid, ethyl ester | C_14_H_28_O_2_ | 228.37 | 73.0, 88.0, 101.0 | 25.51 | 0.6 | - | - | - | - | - | - | - | - | - | - | - | - | - | - | 4.76 | - | - | - |  |
| 107 | 628-97-7 | Hexadecanoic acid, ethyl ester | C_18_H_36_O_2_ | 284.48 | 88.0, 100.9, 206.7 | 32.24 | 2.88 | 2.15 | 4.78 | - | 2.11 | 2.74 | 3.65 | 2.21 | 2.86 | - | 3.69 | 3.85 | 2.02 | 5.2 | 3.74 | 17.13 | 3.37 | - | 2.45 |  |
|  |  | Ketones (16) |  |  |  |  |  |  |  |  |  |  |  |  |  |  |  |  |  |  |  |  |  |  |  |  |
| 108 | 513-86-0 | Acetoin | C_4_H_8_O_2_ | 88.11 | 43.1, 45.1, 61.0 | 2.20 | - | - | - | - | - | - | - | 1.18 | - | - | - | - | - | - | - | 44.18 | - | - | - |  |
| 109 | 823-19-8 | 3-Hydroxycyclohexanone | C_6_H_10_O_2_ | 114.14 | 42.0, 44.0, 57.0 | 5.26 | - | - | - | - | - | - | - | - | - | 0.07 | - | - | - | - | - | - | - | - | - |  |
| 110 | 589-92-4 | 4-Methyl-cyclohexanone | C_7_H_12_O | 112.17 | 43.0, 55.0, 82.9 | 10.86 | - | - | - | - | - | - | - | - | 6.94 | - | - | - | - | - | - | - | - | - | - |  |
| 111 | 585-25-1 | 2,3-Octanedione | C_8_H_14_O_2_ | 142.20 | 43.1, 99.0, 108.0 | 11.28 | - | - | - | - | - | - | - | - | 31.7 | - | - | - | - | - | - | - | - | - | - |  |
| 112 | 110-93-0 | 6-Methyl-5-hepten-2-one | C_8_H_14_O | 126.20 | 43.1, 99.0, 107.9 | 11.31 | 16.08 | - | - | 9.24 | - | - | - | - | - | - | - | 14.67 | - | 13.36 | - | - | - | 18 | - |  |
| 113 | 13905-10-7 | 5-Methyl-4-hexen-3-one | C_7_H_12_O | 112.17 | 55.0, 82.9, 84.0 | 12.22 | 9.14 | 78.01 | - | - | - | 16.84 | - | 3.56 | - | - | - | - | - | - | - | - | - | - | - |  |
| 114 | 6137-06-0 | 4-Methyl-2-heptanone | C_8_H_16_O | 128.21 | 43.0, 58.0, 85.0 | 13.78 | - | - | - | - | - | - | - | - | - | - | 2.33 | - | - | - | - | - | - | - | - |  |
| 115 | 76-22-2 | Camphor | C_10_H_16_O | 152.23 | 41.1, 67.0, 95.0 | 15.58 | - | - | 1.06 | - | - | - | - | - | - | - | - | - | - | - | - | - | - | - | - |  |
| 116 | 7764-50-3 | Dihydrocarvone | C_10_H_16_O | 152.23 | 94.9, 97.0, 136.9 | 20.29 | - | - | - | - | - | - | - | 11.3 | - | - | - | - | - | - | - | - | - | - | - |  |
| 117 | 89-82-7 | Pulegone | C_10_H_16_O | 152.23 | 69.0, 80.9, 96.9 | 20.29 | - | - | - | - | - | - | 8.56 | - | - | - | - | - | - | - | - | - | - | - | - |  |
| 118 | 1000374-18-0 | α-Acorenol | C_15_H_26_O | 222.37 | 119.0, 203.9, 206.8 | 21.01 | - | - | - | - | - | - | - | - | - | - | - | - | - | - | - | - | - | - | 1.58 |  |
| 119 | 127-41-3 | α-Ionone | C_13_H_20_O | 192.30 | 91.0, 92.9, 120.9 | 22.20 | - | 4.82 | - | - | 1.91 | - | - | - | - | - | - | - | - | - | 1.67 | 3.47 | - | - | - |  |
| 120 | 17283-81-7 | Dihydro-beta-ionone | C_13_H_22_O | 194.31 | 92.9 , 121.0, 161.0 | 22.43 | - | 1.22 | - | - | - | - | 1.71 | - | 0.83 | - | - | - | - | - | 1.18 | - | - | - | 1.22 |  |
| 121 | 3796-70-1 | Geranylacetone | C_13_H_22_O | 194.31 | 43.0, 93.0, 136.0 | 22.69 | - | - | - | - | - | - | - | - | - | - | - | 5.75 | 7.68 | - | - | - | - | 8.44 | - |  |
| 122 | 3879-26-3 | Neryl acetone | C_13_H_22_O | 194.31 | 43.1, 107.0, 136.0 | 22.69 | 27.96 | 26.2 | - | - | - | 6.05 | - | - | - | - | - | - | 18.81 | - | - | - | - | 9.8 | - |  |
| 123 | 79-77-6 | β-Ionone | C_13_H_20_O | 192.30 | 91.0, 123.0, 176.9 | 23.43 | 15.57 | 24.87 | 15.36 | 31.99 | 7.15 | 5.74 | 35.07 | 18.04 | 22.72 | 14.92 | 23.08 | 14.17 | 2.53 | 16.18 | 27.5 | 12.38 | - | - | 11.88 |  |
|  |  | Alkenes (36) |  |  |  |  |  |  |  |  |  |  |  |  |  |  |  |  |  |  |  |  |  |  |  |  |
| 124 | 100-42-5 | Styrene | C_8_H_8_ | 104.15 | 51.0, 78.0, 103.9 | 8.46 | - | - | - | - | - | - | - | - | - | - | 375.47 | 458.19 | - | - | - | - | - | - | - |  |
| 125 | 508-32-7 | Cyclene | C_10_H_16_ | 136.23 | 67.0, 81.0, 96.0 | 9.27 | - | - | - | - | - | - | - | - | - | - | - | - | - | - | - | - | 0.3 | - | - |  |
| 126 | 79-92-5 | Camphene | C_10_H_16_ | 136.23 | 67.0, 93.0, 121.0 | 10.09 | - | - | 7.73 | - | - | - | - | - | - | - | - | - | - | - | - | - | - | - | - |  |
| 127 | 16789-51-8 | 3-Ethyl-3-hexene | C_8_H_16_ | 112.21 | 55.0, 83.0, 96.9 | 10.82 | 2.16 | 4.9 | - | - | - | 12.68 | - | - | 9.27 | - | - | - | - | - | - | - | - | - | - |  |
| 128 | 2808-71-1 | 3-Ethylcyclohexene | C_8_H_14_ | 110.20 | 41.1, 67.1, 81.0 | 11.59 | - | - | - | - | - | - | - | - | 5.18 | - | - | - | 3.98 | - | - | - | - | - | - |  |
| 129 | 7785-70-8 | (+)-α-Pinene | C_10_H_16_ | 136.23 | 78.9, 92.9, 105.0 | 12.54 | 12.34 | 31.2 | 12.18 | 16.26 | 8.87 | 16.05 | 13.96 | 12.31 | 12.69 | 32.69 |  | 11.97 | 11.64 | 8.3 | - | 9.26 | 4.13 | - | 15.37 |  |
| 130 | 74752-97-9 | (Z)-3-Ethyl-2-methyl-1,3-hexadiene | C_9_H_16_ | 124.22 | 55.0, 67.0, 94.9 | 12.55 | - | 3.96 | 42.15 | - | - | 28.71 | 19.99 | 33.07 | 18.37 | 50.87 |  | 51.39 | 34.53 | 25.89 | 38.33 | 23 | 14.05 | - | 22.82 |  |
| 131 | 3779-61-1 | trans-.beta.-Ocimene | C_10_H_16_ | 136.23 | 79.0, 91.0, 93.0 | 12.73 | - | 5.59 | 11.7 | - | - | - | - | 27.5 | - | 18.56 | 5.19 | 18.77 | - | - | - | - | - | 2.73 | 7.59 |  |
| 132 | 13466-78-9 | 3-Carene | C_10_H_16_ | 136.23 | 79.0, 92.9, 121.0 | 13.01 | 148.82 | 128.37 | 143.03 | 658.02 | 192.68 | 293.33 | 358.51 | 315.34 | 251.44 | 538.02 | 97.99 | 60.63 | 105.7 | 180.2 | 32.14 | 199.59 | 100.21 | 44.37 | 252.2 |  |
| 133 | 3338-55-4 | (Z)-3,7-dimethylocta-1,3,6,-triene | C_10_H_16_ | 136.23 | 91.0, 93.0, 104.9 | 13.02 | - | - | - | - | - | - | - | - | - | - | 6.4 | 8.68 | - | - | - | - | - | - | - |  |
| 134 | 498-15-7 | (+)-3-Carene | C_10_H_16_ | 136.23 | 76.9, 78.9, 92.9 | 13.25 | - | - | - | 9.88 | - | - | - | - | - | - | - | - | - | - | - | - | - | - | - |  |
| 135 | 824-90-8 | 1-Phenyl-1-butene | C_10_H_12_ | 132.20 | 114.9, 116.0, 131.9 | 14.13 | - | 42.88 | - | - | 24.22 | 10.96 | - | - | - | - | 3.86 | - | - | 20.23 | - | 14.13 | 10.41 | - | 5.91 |  |
| 136 | 768-49-0 | 2-Methyl-1-phenylpropene | C_10_H_12_ | 132.20 | 114.9, 117.0, 131.9 | 14.13 | 46.04 | - | - | 23.76 | 24.83 | - | 17.13 | 38.25 | 28.43 | 41.15 | - | - | - | - | - | 17 | - | - | 27.33 |  |
| 137 | 18368-95-1 | 1,3,8-p-Menthatriene | C_10_H_14_ | 134.22 | 76.97, 90.9, 118.9 | 14.98 | - | 27.13 | 32.59 | 19.37 | 4.65 | 61.3 | 9.39 | 9.21 | 8.97 | 7.64 | - | 21.86 | - | 3.64 | - | - | 13.94 | - | 12.4 |  |
| 138 | 460-01-5 | Cosmene | C_10_H_14_ | 134.22 | 77.0, 90.9, 118.9 | 15.18 | - | - | - | 139.83 | 53.88 | - | 74.18 | 96.19 | 80.38 | 143.46 |  | - | - | 66.93 | - | 65.23 | 58.34 | - | 97.54 |  |
| 139 | 99-86-5 | α-Terpinene | C_10_H_16_ | 136.23 | 76.9, 90.9, 120.9 | 15.19 | - | - | - | - | - | - | - | - | - | - | - | - | 17.02 | - | - | - | - | - | - |  |
| 140 | 3016-19-1 | 2,6-dimethyl-2,4,6-octatriene | C_10_H_16_ | 136.23 | 78.9, 104.9, 120.9 | 15.20 | - | - | - | - | - | - | - | - | - | - | - | - | 73.57 | - | - | - | - | - | - |  |
| 141 | 7216-56-0 | (4E,6Z)-2,6-dimethylocta-2,4,6-triene | C_10_H_16_ | 136.23 | 90.9, 104.9, 120.9 | 15.48 | 13.41 | 21.96 | 11.18 | 57.4 | 18.93 | 15.37 | 34.93 | 38.35 | 30.33 | 51.72 | 8.45 | 8.81 | 10.68 | 14.4 | - | 23.62 | 10.19 | 2.43 | 20.54 |  |
| 142 | 673-84-7 | 2,6-Dimethyl-2,4,6-octatriene | C_10_H_16_ | 136.23 | 79.0, 104.9, 121.0 | 15.48 | - | - | - | - | - | - | - | - | - | - | - | - | - | 18.57 | - | - | - | - | - |  |
| 143 | 14912-44-8 | Ylangene | C_15_H_24_ | 204.35 | 92.9, 119.0, 160.9 | 21.01 | - | - | - | - | - | - | - | - | - | - | - | - | - | - | - | - | 4.3 | - | - |  |
| 144 | 1137-12-8 | Longicyclene | C_15_H_24_ | 204.35 | 94.0, 104.9, 161.0 | 21.02 | 5.17 | 5.78 | 4.45 | 5.1 | - | 1.23 | 5.7 | 20.45 | 2.73 | 8.86 | 8.16 | 6.74 | - | 7.17 | - | - | - | - | 4.59 |  |
| 145 | 17699-14-8 | .alpha.-Cubebene | C_15_H_24_ | 204.35 | 91.0, 119.0, 160.9 | 21.10 | - | - | - | - | - | 4.54 | - | - | - | - | - | - | - | - | - | - | - | - | 29.11 |  |
| 146 | 1000360-33-0 | .alfa.-Copaene | C_15_H_24_ | 204.35 | 92.9, 118.9, 161.0 | 21.11 | - | 4.42 | - | - | 2.75 | - | - | - | - | - | - | - | - | - | - | - | - | 34.75 | 54.06 |  |
| 147 | 3856-25-5 | α-Copaene | C_15_H_24_ | 204.35 | 80.9, 92.9, 118.9 | 21.11 | - | 15.38 | - | - | - | 5.3 | - | 2.61 | - | - | - | - | - | - | - | - | - | - | 5.66 |  |
| 148 | 5989-08-2 | alpha-Longipinene | C_15_H_24_ | 204.35 | 90.8, 92.9, 118.9 | 21.20 | 4.46 | 6.9 | 3.19 | 8.11 | 1.29 | - | 9 | 22.67 | 4.5 | 15.42 | 8.64 | 6.88 | - | 4.7 | - | 3.37 | 2.54 | - | 2.64 |  |
| 149 | 17066-67-0 | beta-Selinene | C_15_H_24_ | 204.35 | 81.1, 108.0, 161.0 | 21.44 | - | - | - | - | - | - | - | - | - | - | - | - | - | - | - | - | - | 6.13 | - |  |
| 150 | 22469-52-9 | Cyclosativene | C_15_H_24_ | 204.35 | 105.0, 118.9, 160.9 | 21.76 | 13.77 | - | - | - | - | - | - | 16.37 | - | - | - | 8.7 | - | 7.42 | 22.31 | 2.19 | - | 10.48 | - |  |
| 151 | 18252-44-3 | beta-Copaene | C_15_H_24_ | 204.35 | 91.0, 133.0, 161.0 | 22.27 | - | - | - | - | - | - | - | - | - | - | - | - | - | - | - | - | - | 1.65 | 0.88 |  |
| 152 | 18252-46-5 | cis-.alpha.-Bergamotene | C_15_H_24_ | 204.35 | 90.9, 93.0, 118.9 | 22.37 | 5.95 | 8.43 | 4.48 | 7.21 | 4.53 | - | 5.3 | 14.55 | 2.97 | 4.18 | - | 9.01 | 9.67 | 3.97 | 8.93 | - | - | - | 4.27 |  |
| 153 | 52254-38-3 | 2-Methyl-1-tetradecene | C_15_H_30_ | 210.40 | 41.1, 56.0, 57.1 | 22.50 | - | - | - | 5.47 | - | - | - | 12.08 | - | - | - | - | - | - | - | - | - | - | - |  |
| 154 | 13877-93-5 | Caryophyllene | C_15_H_24_ | 204.35 | 43.1, 69.0, 93.0 | 22.70 | - | - | 13.75 | - | - | - | - | - | - | - | - | - | - | - | - | - | - | - | - |  |
| 155 | 18431-82-8 | beta-Chamigrene | C_15_H_24_ | 204.35 | 79.0, 93.9, 160.9 | 22.72 | - | 69.2 | - | 20.36 | - | - | 31.24 | - | - | - | - | - | - | - | - | - | - | - | - |  |
| 156 | 53111-25-4 | gamma-Himachalene | C_15_H_24_ | 204.35 | 92.9, 119.0, 132.9 | 23.32 | - | - | - | 28.32 | - | 2.09 | 37.38 | 76.37 | 21.6 | 47.63 | 21.7 | 7.28 | - | - | 56.75 | 8.83 | 13.74 | 2.46 | 13.53 |  |
| 157 | 1461-03-6 | beta-Himachalene | C_15_H_24_ | 204.35 | 91.0, 104.9, 118.9 | 24.21 | 4.47 | 13.26 | 4.99 | 24.13 | 1.52 | 2 | 21.61 | 45.42 | 12.63 | 31.32 | 21.42 | 31.88 | - | 10.14 | - | 5.12 | 9.18 | - | 7.01 |  |
| 158 | 19419-67-1 | aR-Himachalene | C_15_H_22_ | 202.34 | 144.9, 187, 201.9 | 24.52 | - | - | - | - | - | - | - | 8.05 | - | 5.28 | - | - | - | - | - | - | 5.6 | - | - |  |
| 159 | 14811-95-1 | 1,19-Eicosadiene | C_20_H_38_ | 278.52 | 81.0, 82.1, 94.9 | 25.14 | - | 6.6 | - | - | - | - | - | - | - | 3.81 | - | - | - | - | - | - | - | - | 1.44 |  |
|  |  | Alkane (18) |  |  |  |  |  |  |  |  |  |  |  |  |  |  |  |  |  |  |  |  |  |  |  |  |
| 160 | 108-87-2 | Methylcyclohexane | C_7_H_14_ | 98.19 | 58.0, 69.0, 83.0 | 7.43 | - | 300.08 | 264.8 | - | 376.61 | 439.3 | 91.89 | - | 682.13 | 372.42 | 213.76 | 220.3 | 433.7 | 351.67 | 234.56 | 390.62 | 135.86 | 469.03 | - |  |
| 161 | 3178-22-1 | tert-Butylcyclohexane | C_10_H_20_ | 140.27 | 41.1, 56.1, 69.0 | 7.84 | - | - | - | - | - | - | - | - | - | - | - | - | - | - | 147.97 | - | - | - | - |  |
| 162 | 2207-01-4 | 1,2-cis-Dimethylcyclohexane | C_8_H_16_ | 112.21 | 55.0, 70.0, 83.0 | 10.85 | - | 2.03 | - | - | - | - | - | - | - | - | - | - | - | - | - | 6.07 | - | - | - |  |
| 163 | 629-50-5 | Tridecane | C_13_H_28_ | 184.36 | 43.1, 57.1, 71.0 | 19.30 | 4.4 | - | - | - | - | 23.91 | - | - | - | - | - | - | - | - | - | - | - | - | 4.18 |  |
| 164 | 565-59-3 | 2,3-Dimethylpentane | C_7_H_16_ | 100.20 | 43.0, 56.0, 71.0 | 20.57 | - | - | - | - | - | - | - | - | - | - | 0.14 | - | - | - | - | - | - | - | - |  |
| 165 | 1560-96-9 | 2-Methyltridecane | C_14_H_30_ | 198.39 | 57.1, 71.0, 85.0 | 20.72 | 16.48 | 60.77 | - | 12.64 | - | - | 14.15 | 18.21 | 8.89 | 19.2 | 20.31 | 5.2 | 4.32 | 6.13 | 42.86 | 11.23 | - | - | 12.08 |  |
| 166 | 56862-62-5 | 10-Methylnonadecane | C_20_H_42_ | 282.55 | 57.0, 71.0, 99.1 | 20.72 | 9.83 | - | 9.63 | 8.52 | 4.46 | - | - | - | - | - | - | - | - | 9.36 | - | - | - | - | 3.35 |  |
| 167 | 294-62-2 | Cyclododecane | C_12_H_24_ | 168.32 | 69.0, 83.0, 97.0 | 21.35 | 5.91 | 6.5 | - | - | - | - | - | - | - | - | - | - | - | - | - | - | - | - | - |  |
| 168 | 295-17-0 | Cyclotetradecane | C_14_H_28_ | 196.37 | 55.0, 69.0, 111.0 | 21.35 | - | - | - | - | 19.12 | - | - | - | - | - | - | - | - | - | - | - | - | 6.05 | - |  |
| 169 | 629-59-4 | Tetradecane | C_14_H_30_ | 198.39 | 43.0, 71.0, 85.1 | 21.52 | 6.46 | 3.26 | - | - | 3.36 | 7.27 | - | - | - | - | - | - | - | - | 4.73 | - | - | 2.26 | - |  |
| 170 | 295-48-7 | Cyclopentadecane | C_15_H_30_ | 210.40 | 44.1, 69.0, 97.0 | 22.50 | - | 18.02 | - | - | - | - | 7.2 | 18.5 | - | - | - | - | - | - | 18.71 | - | - | - | - |  |
| 171 | 20959-33-5 | 7-Methylheptadecane | C_18_H_38_ | 254.49 | 57.1, 71.0, 169.1 | 22.85 | - | - | 4.86 | - | - | - | - | - | - | - | - | - | - | - | - | - | - | - | - |  |
| 172 | 1560-95-8 | 2-Methyltetradecane | C_15_H_32_ | 212.42 | 57.0, 71.0, 85.1 | 22.86 | 5.12 | 17.72 | 6.72 | 6.73 | - | - | 6.74 | 14.96 | - | 6.26 | 7.03 | 7.54 | - | 5.4 | 23.82 | 4.56 | - | - | 3.98 |  |
| 173 | 504-44-9 | 2,6,11,15-Tetramethylhexadecane | C_20_H_42_ | 282.55 | 43.1, 57.1, 169.0 | 22.86 | - | - | - | 5.11 | 3.16 | - | - | - | - | - | 5.06 | 4.67 | - | - | - | 5.13 | - | - | 3.78 |  |
| 174 | 18435-22-8 | 3-Methyl tetradecane | C_15_H_32_ | 212.42 | 57.0, 71.0, 183.2 | 24.87 | - | - | - | - | - | - | - | - | - | - | 2.61 | - | - | - | - | 3.01 | - | - | - |  |
| 175 | 1560-93-6 | 2-Methylpentadecane | C_16_H_34_ | 226.44 | 57.0, 71.0, 85.0 | 24.87 | 1.99 | 12.31 | 2.99 | 1.88 | - | - | 2.94 | 3.15 | - | 5.2 | 3.41 | - | - | - | 9.02 | - | - | - | - |  |
| 176 | 544-76-3 | Hexadecane | C_16_H_34_ | 226.44 | 29.0, 71.0, 85.0 | 25.59 | 2.68 | - | - | - | - | - | - | 1.84 | - | 3.44 | - | 4.88 | - | - | 6.24 | - | - | - | - |  |
| 177 | 629-78-7 | Heptadecane | C_17_H_36_ | 240.47 | 57.1, 71.0, 113.0 | 27.47 | - | 3.02 | - | 3.82 | - | - | 2.46 | 4.36 | - | - | 3.29 | 3.88 | - | - | 1.98 | - | - | - | - |  |
|  |  | Acids (6) |  |  |  |  |  |  |  |  |  |  |  |  |  |  |  |  |  |  |  |  |  |  |  |  |
| 178 | 144-62-7 | Oxalic acid | C_2_H_2_O_4_ | 90.04 | 43.1, 45.1, 46.1 | 1.55 | - | 48.94 | - | - | - | - | 36.35 | 16.67 | - | - | - | - | - | - | - | - | - | - | - |  |
| 179 | 64-19-7 | Acetic acid | C_2_H_4_O_2_ | 60.05 | 45.0, 60.1, 81.0 | 4.26 | - | - | 8.82 | 26.65 | - | - | - | - | - | - | 0.52 | - | - | - | - | - | - | - | - |  |
| 180 | 54699-35-3 | 1,2,4-Benzenetricarboxylic acid | C_11_H_10_O_6_ | 238.19 | 41.1, 56.1, 206.8 | 6.91 | - | - | - | - | - | - | - | - | - | - | - | - | - | - | - | 0.91 | - | - | - |  |
| 181 | 142-62-1 | Hexanoic acid | C_6_H_12_O_2_ | 116.16 | 60.0, 80.9, 86.9 | 14.08 | 158.86 | - | - | - | - | - | - | - | - | - | - | - | - | - | - | - | - | - | - |  |
| 182 | 109-52-4 | Pentanoic acid | C_5_H_10_O_2_ | 102.13 | 55.0, 61.0, 73.0 | 15.85 | - | - | - | - | - | - | - | - | - | - | 1.27 | - | - | - | - | - | - | - | - |  |
| 183 | 124-07-2 | Octanoic acid | C_8_H_16_O_2_ | 144.21 | 55.0, 60.0, 73.0 | 20.28 | 11.32 | - | - | - | - | - | - | - | - | - | - | - | - | - | - | - | - | - | - |  |
|  |  | Others (16) |  |  |  |  |  |  |  |  |  |  |  |  |  |  |  |  |  |  |  |  |  |  |  |  |
| 184 | 75-09-2 | Methylene chloride | CH_2_Cl_2_ | 84.93 | 44.1, 45.1, 84.0 | 2.19 | - | - | - | - | - | - | - | - | - | - | - | - | 101.86 | - | - | - | - | - | - |  |
| 185 | 544-10-5 | Chlorohexane | C_6_H_13_Cl | 120.62 | 55.0, 70.1, 90.9 | 4.91 | - | - | - | 2.1 | - | - | - | - | 5.08 | - | - | - | - | - | - | - | - | - | - |  |
| 186 | 108-88-3 | Toluene | C_7_H_8_ | 92.14 | 42.1, 57.0, 91.0 | 4.96 | - | - | - | - | - | 9.02 | - | - | - | - | 156.36 | 151.84 | - | - | - | - | - | - | - |  |
| 187 | 95-47-6 | o-Xylene | C_8_H_10_ | 106.17 | 82.0, 90.9, 105.9 | 7.73 | - | - | - | - | - | - | - | - | - | - | - | 52.39 | - | - | - | - | - | - | - |  |
| 188 | 106-42-3 | p-Xylene | C_8_H_10_ | 106.17 | 56.1, 69.0, 90.9 | 7.86 | - | - | - | - | - | - | - | - | - | - | - | 416.41 | - | - | - | - | - | - | - |  |
| 189 | 3208-16-0 | 2-Ethylfuran | C_6_H_8_O | 96.13 | 67.0, 80.9, 96.0 | 8.84 | 32.35 | 63.17 | 34.63 | 9.34 | 25.26 | 56.67 | 27.15 | 44.48 | 54.94 | 9.55 |  | 18.81 | 23.81 | 18.53 | 69.38 | 35.49 | 7.68 | 14.86 | 14.19 |  |
| 190 | 286-45-3 | 8-Oxabicyclo[5.1.0]octane | C_7_H_12_O | 112.17 | 68.0, 82.9, 105.9 | 10.40 | - | - | 20.38 | - | 63.26 | - | - | - | - | - |  | - | - | - | - | - | - | - | 18.23 |  |
| 191 | 3777-69-3 | 2-Pentylfuran | C_9_H_14_O | 138.21 | 43.1, 58.0, 80.9 | 11.42 | 18.07 | - | 119.11 | 30.98 | - | - | - | - | 43.6 | 127.74 |  | - | - | 56.43 | 101.06 | - | 69.34 | 8.82 | 69.68 |  |
| 192 | 99-87-6 | p-Cymene | C_10_H_14_ | 134.22 | 90.9, 118.9, 120.0 | 12.37 | - | - | 1.85 | 8.16 | 0.13 | - | 1.68 | - | 1.35 | 2.42 | - | - | 0.78 | 1.26 | - | - | 0.86 | - | - |  |
| 193 | 24683-00-9 | 2-Isobutyl-3-methoxypyrazine | C_9_H_14_N_2_O | 166.22 | 93.9, 123.9, 150.9 | 16.56 | 163.43 | 164.46 | 238.49 | 265.36 | 179.02 | 165.48 | 275.61 | 267.3 | 312.41 | 267.94 | 183.67 | 172 | 307.03 | 246.71 | 328.92 | 233.2 | 249.7 | 223.75 | 203.5 |  |
| 194 | 108-95-2 | Phenol | C_6_H_6_O | 94.11 | 65.0 66.0, 94.0 | 17.22 | 36.92 | - | - | - | - | - | - | - | - | - | 0.41 | - | - | - | - | - | - | - | - |  |
| 195 | 90-05-1 | Guaiacol | C_7_H_8_O_2_ | 124.14 | 81.0, 95.0, 109.0 | 17.56 | 124.11 | - | - | - | - | - | - | - | - | - | 1.19 | - | - | - | - | - | - | - | - |  |
| 196 | 58334-55-7 | Zingiberenol | C_15_H_26_O | 222.37 | 69.1, 135.9, 150.9 | 22.70 | - | - | - | - | - | - | - | - | - | - | - | - | - | - | 9.74 | - | - | - | - |  |
| 197 | 629-89-0 | 1-Octadecyne | C_18_H_34_ | 250.46 | 43.1, 68.0, 80.9 | 23.47 | - | 2.79 | - | - | 7.36 | - | - | - | - | 5.24 | 6.99 | - | - | - | 3.95 | - | - | - | - |  |
| 198 | 1138-52-9 | 3,5-Di-tert-butylphenol | C_14_H_22_O | 206.32 | 57.0, 190.9, 205.9 | 23.91 | - | - | 2.35 | - | - | - | - | - | - | - | - | - | - | - | - | - | - | - | - |  |
| 199 | 128-37-0 | Butylated Hydroxytoluene | C_15_H_24_O | 220.35 | 204.9, 205.9, 219.8 | 23.96 | 0.4 | 0.87 | - | - | - | 16.19 | 2.03 | - | - | - | - | - | - | - | - | - | - | - | - |  |

Note: ^a^ Compound number. ^b^ Chemical Abstracts Services Registry number. ^c^ Compounds in order of their retention time in a chemical class. ^d^ Molecular formula. ^e^ Molecular weight. ^f^ The characteristic ion (m/z) was employed for selecting the corresponding compound in order to avoid possible interference by other volatiles. ^g^ Retention time. ^h^ Each value is the mean of triplicate biological samples taken from the same varieties; “-”, not detected. W1, Fengshouxianjiao; W2, Sujiao 8; W3, Qingan 7; W4, Sujiao 18; W5, Longjiao 2; W6, Longjiao 10; W7, Longjiao 11; W8, Huamei 105; W9, 37-124; W10, 3F*106; W11, Tianjiao 20; W12, Tianjiao 23; W13, Hangjiao 2; W14, Hangjiao 8; W15, NO.171; S1, NO.212; S2, NO.221; S3, Sujiao 9; S4, HJF42.

Supplementary Table 3 Odor activity values of 14 volatile compounds in from 19 pepper varieties

| CAS number | Compounds name | Odor description | Odor threshold | Odor activity values (OAVs) | | | | | | | | | | | | | | | | | | |
| --- | --- | --- | --- | --- | --- | --- | --- | --- | --- | --- | --- | --- | --- | --- | --- | --- | --- | --- | --- | --- | --- | --- |
|  |  |  | μg/kg | W1 | W2 | W3 | W4 | W5 | W6 | W7 | W8 | W9 | W10 | W11 | W12 | W13 | W14 | W15 | S1 | S2 | S3 | S4 |
| 7212-44-4 | Nerolidol | Floral, waxy, citrus | 10 | - | 0.20 | - | - | - | - | - | - | - | - | - | - | - | - | - | - | - | - | - |
| 928-94-9 | (Z)-2-hexen-1-ol | Green, cortex, bean | 359 | - | - | - | - | 0.59 | - | - | - | - | - | - | - | - | - | - | - | - | - | - |
| 124-25-4 | Tetradecanal | Fatty, waxy, incense | 60 | 0.38 | 0.07 | - | - | 0.11 | - | - | 0.12 | - | 0.07 | 0.28 | 0.21 | - | - | 0.19 | 0.14 | - | 0.16 | - |
| 105-57-7 | 1,1-Diethoxy-ethane | Green, nut, earthy | 100 | 0.04 | 0.17 | 0.10 | 0.03 | 0.01 | 0.18 | 0.04 | 0.15 | - | 0.04 | 0.06 | 0.03 | 0.09 | 0.10 | 0.01 | 0.36 | 0.22 | 0.14 | 0.06 |
| 93-89-0 | Benzoic acid, ethyl ester | Fruity, wintergreen | 60 | - | 0.33 | - | 0.18 | - | - | 0.45 | 0.34 | 0.39 | - | - | 0.29 | - | - | - | 0.19 | - | - | - |
| 110-93-0 | 6-Methyl-5-hepten-2-one | Lemongrass, apple | 50 | 0.32 | - | - | 0.18 | - | - | - | - | - | - | - | 0.29 | - | 0.27 | - | - | - | 0.36 | - |
| 3779-61-1 | β.-Ocimene | Sweet, herbal | 34 | - | 0.16 | 0.34 | - | - | - | - | 0.81 | - | 0.55 | 0.15 | 0.55 | - | - | - | - | - | 0.08 | 0.22 |
| 13466-78-9 | 3-Carene | Citrus, herbal, pine | 770 | 0.19 | 0.17 | 0.19 | 0.85 | 0.25 | 0.38 | 0.47 | 0.41 | 0.33 | 0.70 | 0.13 | 0.08 | 0.14 | 0.23 | 0.04 | 0.26 | 0.13 | 0.06 | 0.33 |
| 99-86-5 | α-Terpinene | Woody, lemon, herbal | 85 | - | - | - | - | - | - | - | - | - | - | - | - | 0.20 | - | - | - | - | - | - |
| 14912-44-8 | Ylangene | - | 6 | - | - | - | - | - | - | - | - | - | - | - | - | - | - | - | - | 0.72 | - | - |
| 100-42-5 | Styrene | Sweet, balsam, floral | 730 | - | - | - | - | - | - | - | - | - | - | 0.51 | 0.63 | - | - | - | - | - | - | - |
| 13877-93-5 | Caryophyllene | Sweet, woody, spice | 64 | - | - | 0.21 | - | - | - | - | - | - | - | - | - | - | - | - | - | - | - | - |
| 142-62-1 | Hexanoic acid | Sour, sweat, cheese | 200 | 0.79 | - | - | - | - | - | - | - | - | - | - | - | - | - | - | - | - | - | - |
| 106-42-3 | p-Xylene | - | 450.5 | - | - | - | - | - | - | - | - | - | - | - | 0.92 | - | - | - | - | - | - | - |

Note: Aroma characteristic referenced in http://www.thegoodscentscompany.com/. -, not detected. W1, Fengshouxianjiao; W2, Sujiao 8; W3, Qingan 7; W4, Sujiao 18; W5, Longjiao 2; W6, Longjiao 10; W7, Longjiao 11; W8, Huamei 105; W9, 37-124; W10, 3F*106; W11, Tianjiao 20; W12, Tianjiao 23; W13, Hangjiao 2; W14, Hangjiao 8; W15, NO.171; S1, NO.212; S2, NO.221; S3, Sujiao 9; S4, HJF42.
